# A scoping review of preclinical intensive care unit-acquired weakness models

**DOI:** 10.3389/fphys.2024.1423567

**Published:** 2024-10-02

**Authors:** Qingmei Yu, Jiamei Song, Luying Yang, Yanmei Miao, Leiyu Xie, Xinglong Ma, Peng Xie, Shaolin Chen

**Affiliations:** ^1^ Nursing Department, Affiliated Hospital of Zunyi Medical University, Zunyi, Guizhou, China; ^2^ School of Nursing, Zunyi Medical University, Zunyi, Guizhou, China; ^3^ Department of Critical Care Medicine, The Third Affiliated Hospital of Zunyi Medical University, The First People’s Hospital of Zunyi City, Zunyi, Guizhou, China; ^4^ Department of Critical Care Medicine, The Second Affiliated Hospital, Hengyang Medical School, University of South China, Hengyang, Hunan, China

**Keywords:** intensive care unit-acquired weakness, animal model, muscle weakness, muscle atrophy, scoping review

## Abstract

**Background:**

Animal models focusing on neuromuscular outcomes are crucial for understanding the mechanisms of intensive care unit-acquired weakness (ICU-AW) and exploring potential innovative prevention and treatment strategies.

**Aim:**

To analyse and evaluate preclinical ICU-AW models.

**Methods:**

We manually searched five English and four Chinese databases from 1 January 2002, to 1 February 2024, and reviewed related study references. Full-text publications describing animal models of muscle weakness and atrophy in critical illness were included. Detailed information about model types, animal species, sex, age, induction methods, outcome measures, drawbacks and strengths was extracted from each included study.

**Results:**

A total of 3,451 citations were initially retrieved, with 84 studies included in the final analysis. The most frequently studied animal model included rodents (86.9%), 64.3% of which were male animals. ICU-AW animal models were mostly induced by comprehensive intensive care unit (ICU) interventions (38.1%) and sepsis (51.2%). Most studies focused on limb muscles (66.7%), diaphragm muscles (21.4%) or both (9.5%). Reported outcomes primarily included muscular pathological changes (83.3%), electrophysiological examinations of muscles (57.1%) and animal grip strength (16.6%). However, details such as animal age, mortality data, experimental design, randomisation, blinding, sample size and interventions for the experimental group and/or control group were inadequately reported.

**Conclusion:**

Many preclinical models are used to study ICU-AW, but the reporting of methodological details is often incomplete. Although current ICU animal models can mimic the characteristics of human ICU-AW, there is no standard model. Future preclinical studies should develop a standard ICU-AW animal model to enhance reproducibility and improve scientific rigor in exploring the mechanisms and potential treatment of ICU-AW.

## Introduction

As the number of intensive care unit (ICU) survivors increases, ICU-acquired weakness (ICU-AW) has become a common neuromuscular complication (Kress and Hall, 2014), with an overall incidence rate of 48% (Fazzini et al., 2023). ICU-AW is characterised by the acute onset of generalised symmetrical muscle weakness affecting the extremities and respiratory muscles in critically ill patients. It typically develops following prolonged ICU stays and cannot be attributed to the original disease (Lad et al., 2020). This weakness is often accompanied by muscle wasting and manifests alone or in combination with polyneuropathy, myopathy and muscle atrophy (Piva et al., 2019; Gonzalez et al., 2022). ICU-AW has three subtypes: critical illness polyneuropathy (CIP), critical illness myopathy (CIM) and critical illness neuromyopathy (CINM). CIP involves the peripheral nervous system, CIM affects the muscles, and CINM involves both muscles and nerves (Intiso et al., 2022).

An ICU-AW diagnosis is based on clinical features, neurophysiological testing and nerve and muscle biopsies. Clinically, ICU-AW is characterised by symmetrical weakness with a Medical Research Council score <48. Neurophysiological testing can help differentiate the subtypes of ICU-AW. CIP is indicated by distal sensory loss, reduced compound muscle action potentials (CMAP) and sensory nerve action potentials (SNAP), with normal or near-normal nerve conduction velocities. CIM is identified by short-duration, low-amplitude motor unit potentials on electromyography (EMG), reduced CMAP on direct muscle stimulation and muscle biopsy showing atrophy with thick filament loss or necrosis (Sapra, 2021). CINM combines the characteristics of both CIP and CIM (Wieske et al., 2011).

ICU-AW lacks a discernible underlying cause other than the critical illness itself. It is often associated with the high severity of illness upon admission, sepsis and/or shock, multiple organ dysfunction syndrome (MODS), mechanical ventilation (MV), prolonged sedation and immobilisation, hyperglycaemia, corticosteroid use, neuromuscular blocking agent application and older age in patients (Elkalawy et al., 2023).

Muscle atrophy and loss of muscle function associated with critical illness contribute to difficulties in ventilation weaning, prolong mechanical duration, extend ICU and hospital stay, increase morbidity and mortality within and outside the hospital and even reduce long-term physical function and quality of life (Sapra, 2021). Therefore, understanding the pathogenesis of ICU-AW, particularly at the molecular level, is an urgent concern. This knowledge is crucial for developing future therapies to prevent ICU-AW and facilitate recovery (Lad et al., 2020).

However, the mechanisms underlying ICU-AW are intricate, involving functional and structural alterations in the muscles and nerves (Hermans, 2015). Muscle biopsy and muscle electrophysiological examination are two crucial components in exploring the pathogenesis and molecular mechanisms of critically ill patients experiencing ICU-AW. Nevertheless, the utilisation of these methods presents ethical challenges and is associated with several complications, including pain, bleeding and nerve damage. These conditions are caused by the invasive nature of the methods, which contributes to the difficulty of their implementation in clinical practice (Yoshihara et al., 2023).

In this context, the establishment of an appropriate animal model for ICU-AW is crucial to offer insights into the pathophysiology of muscular and neurological injuries during the development of ICU-AW (Guillon et al., 2019; Liu et al., 2023; Addinsall et al., 2022). However, the capacity of current models to accurately reflect the characteristics of muscular weakness and atrophy in ICU-AW and their ability to detect the effects of triggering on outcomes remain unclear.

This scoping review aims to comprehensively analyse and evaluate the characteristics of current animal models of ICU-AW as well as provide detailed methodologies and assessment of neuromuscular outcomes. Moreover, it seeks to analyse the advantages and disadvantages associated with each model type and recommend the most suitable model for future preclinical research based on clinical practice.

## Materials and methods

This scoping review was conducted in accordance with the Preferred Reporting Items for Systematic Reviews and Meta-Analyses extension for Scoping Reviews (Tricco et al., 2018). The review procedures, including literature retrieval, screening, selection, data extraction and summarisation, were independently carried out by two authors (YQM and SJM). Any disagreements were resolved through consensus or consultation with a third senior author (CSL).

### Eligibility criteria

An ICU-AW animal model involves intentionally inducing the illness in animals to produce subsequent related muscular weakness and atrophy (Zou et al., 2018). The eligibility criteria encompass the following: (1) Population: all animal experimental models of ICU-AW or those investigating critical illness impacting muscular weakness and atrophy; (2) Intervention: any triggered intervention referring to clinical etiological factors inducing ICU-AW during the course of critical illness; (3) Controls: animals compared with the intervention group without triggered factors or other interventions or before-and-after controls; (4) Outcomes: any outcome associated with muscular weakness or atrophy; (5) Literature type: original research; and (6) Languages: English or Chinese.

The exclusion criteria are as follows: (1) research topic is unrelated to ICU-AW; (2) cell culture, human experimentation or use of baby animals; (3) target tissue is not muscle; (4) non-neuromuscular outcomes; (5) review or commentary articles and conference abstracts, and (6) studies with unavailable full text.

### Search strategy

Given that ICU-AW was first studied in a clinical setting in 2002 (De Jonghe et al., 2002), our search period ranged from 1 January 2002, to 1 February 2024. The following databases were searched: Medline, PubMed, Web of Science, Embase, Cochrane Library, China National Knowledge Infrastructure (CNKI), China Science and Technology Journal Database (VIP databases), SinoMed and Wanfang database. The search strategy was developed in collaboration with trained medical librarians and included keywords aligned with the inclusion criteria. Reference lists from relevant reviews and included studies were likewise checked. The detailed search strategy is presented in Supplementary Table S1.

### Study selection and data extraction

The literature review and data synthesis were conducted in March 2024. EndNote X9 (Clarivate Analytics, Philadelphia, PA, United States) was employed for literature management and screening. After duplicate articles were removed, the titles and abstracts of the remaining literature were reviewed. Subsequently, the full text of potential studies was meticulously reviewed to decide the final inclusion.

In this study, ICU-AW models are defined as those in which certain methods are used to induce animals to mimic the complex clinical conditions of patients to research the pathogenesis of the ICU-AW and targets for intervention (Zou et al., 2018). A standardized data extraction form was developed following thorough discussions within our research team and was tested prior to the formal data collection. Extracted data encompassed authors, publication year and country, study objective and design, type of animal model, animal characteristics, induction of animal models, utilisation of life-supporting treatment, sample size, outcomes and time-point of outcome measurements, and drawbacks and strengths from each included study. The collected outcomes primarily included changes in muscle function, morphology, muscle atrophy markers, muscle electrophysiological measurements, body weight, muscle weight, muscle strength, mortality, behavioural changes and other relevant indicators. Categorical variables were presented as numbers and percentages. Continuity variables for abnormal distribution are expressed as median and their interquartile range.

## Results

### Selection process

**FIGURE 1:**
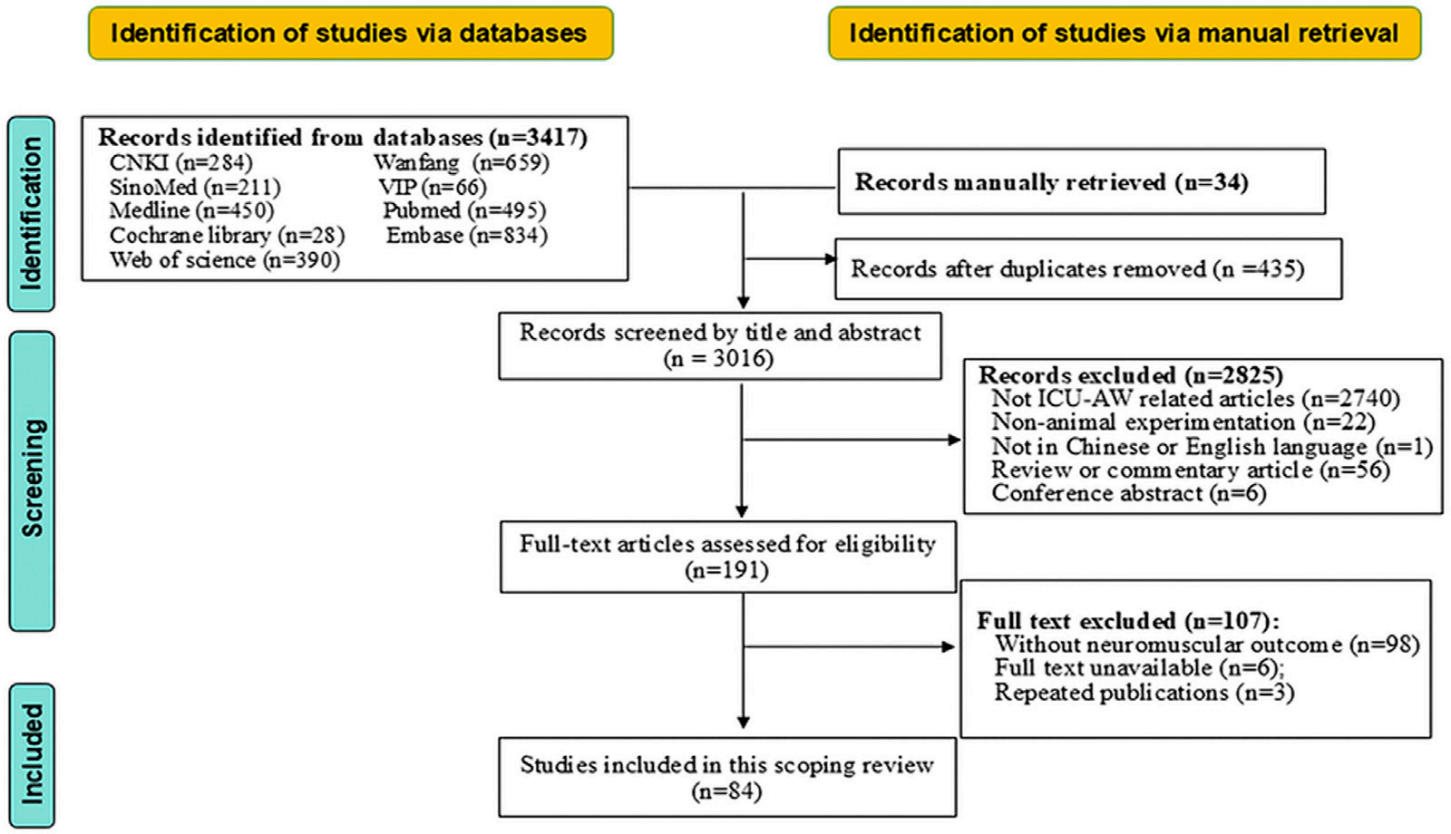
Flow diagram of search screening.

A total of 3,451 citations were initially retrieved. Ultimately, 84 studies were included in this review, including three in Chinese. The detailed flow chart of study selection is displayed in Figure 1.

### Characteristics of included studies

The characteristics of ICU-AW animal models are summarised in Supplementary Table S2. Nearly half of all publications (46.4%, n = 39/84) were released in the recent 5 years. Studies were conducted across 13 countries, of which the top three original countries were the United States, China and Sweden (Table 1).

**TABLE 1:** Characteristics of included studies.

Variables	Total (n = 84)
Year of publication	
2020–2023	31 (36.9%)
2014–2019	31 (36.9%)
2008–2013	17 (20.2%)
2002–2007	5 (6.0%)
Original Country	
USA	20 (23.8%)
China	20 (23.8%)
Sweden	12 (14.3%)
France	7 (8.3%)
Belgium	5 (6.0%)
Japan	7 (8.3%)
Germany	4 (4.8%)
Netherlands	3 (3.6%)
British	2 (2.4%)
Mexico	1 (1.2%)
Turkey	1 (1.2%)
Italy	1 (1.2%)
Jordan	1 (1.2%)
Objective	
Exploring mechanism	51 (60.7%)
Physical interventions	6 (7.1%)
Pharmaceutical or others interventions	20 (23.8%)
Developing ICU-AW animal model	7 (8.3%)
Animal species	
Mice	38 (45.2%)
Rats	35 (41.7%)
Pigs	10 (11.9%)
Rabbits	1 (1.2%)
Sexs	
Male	54 (64.3%)
Female	15 (17.9%)
Both	3 (3.6%)
Animal age	
Juvenile	30 (35.7%)
Adults	25 (29.8%)
Baby and elder	1 (1.2%)
Target muscle	
Diaphragm	18 (21.4%)
Limb muscle	56 (66.7%)
Diaphragm and limb muscle	8 (9.5%)
Masseter Muscle	1 (1.2%)
Masseter Muscle and limb muscle	1 (1.2%)
Mortality reported	
Yes	36 (42.9%)
No	48 (57.1%)
Life-support treatment after induction	
Yes	60 (71.4%)
No	24 (28.6%)

Table 1 presents the characteristics of all included studies. The most common objective of the included studies was exploring ICU-AW pathogenesis (60.7%, n = 51/84). Rodents, primarily mice (45.2%, n = 38/84) and rats (41.7%, n = 35/84), were used, and the most common species were C57 BL/6 mice (39.3%,n = 33/84) and Sprague Dawley rats (26.2%, n = 22/84). Notably, 64.3% of the studies (n = 54/84) used male animals, and only 17.9% used female animals (n = 15/84). Only 56 studies (66.7%) described the animal age.

A total of 46.4% of the studies reported utilising randomised controlled trials (RCTs) (n = 39/84). However, none of these studies specified the exact methods of randomisation. Specifically, 4.8% did not specify the use of a control group (n = 4/84), and none reported blinding. Only one study estimated the sample size, which was described vaguely in 22 studies (26.2%). The median number of animals used in the remaining studies for the intervention group and the control group was 8 (Intiso et al., 2022; Yoshihara et al., 2023) and 8 (Intiso et al., 2022; Tricco et al., 2018), respectively.

**FIGURE 2:**
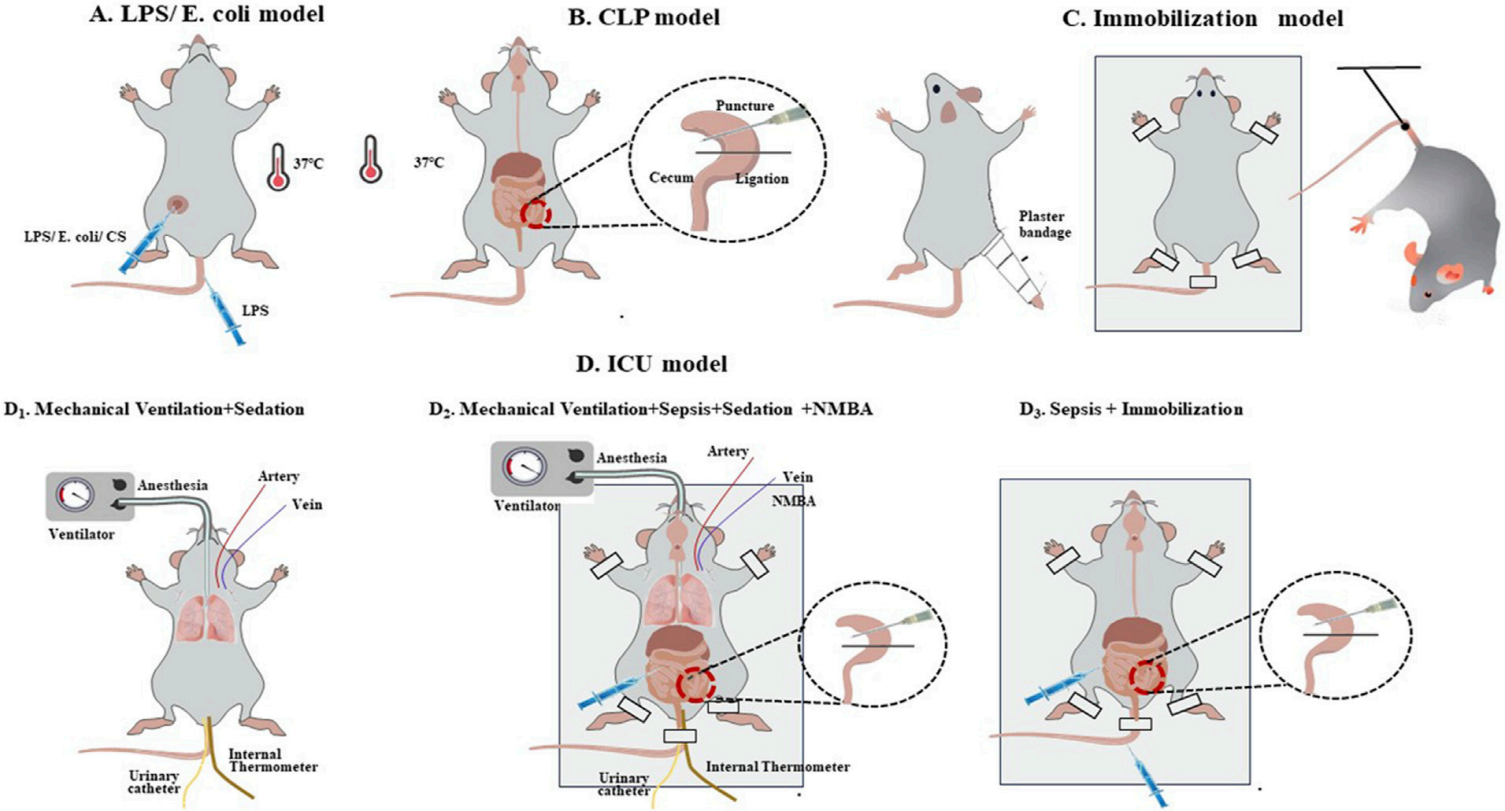
Common induction methods for the intensive care unit-acquired weakness animal model. **(A, B)**. Sepsis models; **(C)**. Immobilisation model; **(D)**: ICU model: D_1_. Mechanical Ventilation + Sedation; D_2_. Mechanical Ventilation + Sepsis + Sedation + NMBA; D_3_. Sepsis + Immobilisation.

Regarding model categorisation, sepsis models, comprising 51.2% of the total (n = 43/84), were predominantly induced by caecal ligation and perforation (CLP) (67.4%, n = 29/43), followed by intraperitoneal injection of lipopolysaccharides (LPS) (14.0%, n = 6/43) or *Escherichia coli* (4.7%, n = 2/43) (Figures 2A, B; Table 2). The second most common models were ICU models (38.1%, n = 32/84), which are those that replicate the complex clinical conditions of human ICU patients with multiple risk factors, including severe sepsis, immobilisation, MV, MODS, hyperglycaemia, denervation, steroids and/or neuromuscular blockade drug usage, and so on (Lad et al., 2020) (Figure 2D, Table 2). Most ICU models (78.1%, n = 25/32) were typically triggered by MV after deep anaesthesia or sedation, with or without other ICU-AW risk factors, such as neuromuscular blockers agent (NMBA), sepsis or corticosteroids (CS) and life support treatment and monitoring (Figure 2D_1–2_; Table 2). Other ICU models mostly included sepsis with immobilisation (15.6%, n = 5/32) (Figure 2D_3_; Table 2). Furthermore, 4.8% of the included studies were disuse models (n = 4/84; Figure 2C; Table 2).

**TABLE 2:** Characteristics of different ICU-AW animal models.

Type	Induction methods	N (%)	Strengths	Drawbacks	Included studies
Sepsis model	CLP	29 (67.4)	Can adjust for sepsis severity	Complex and invasive process, the model is susceptible to death due to infection	Nardelli et al. (2016), Zhang et al. (2016), Goossens et al. (2021), Jude et al. (2020), Alamdari et al. (2012), Liu et al. (2023), Zheng et al. (2023), Weckx et al. (2022), Schmitt et al. (2023), Crowell and Lang (2021), Rocheteau et al. (2015), Vankrunkelsven et al. (2022), Cao et al. (2021), Wang and Wu (2020), Hou et al. (2023), Hou et al. (2021), Li et al. (2023), Chen et al. (2023), Hahn et al. (2020), Nardelli et al. (2013), Zanders et al. (2022), Wang et al. (2023), Supinski et al. (2020a), Supinski et al. (2014), Cankayali et al. (2007), Rossignol et al. (2008), Supinski et al. (2020b), Liu et al. (2014), Vankrunkelsven et al. (2023)
	IP. Or IV. LPS/*E. coli*/Caecal slurry/Zymosan/IN. S. pneumoniae	14 (32.6)	Simple and has a low error rate compared to the CLP mode	Controversial usage due to short-term inflammation and without infected lesions	Chen et al. (2023), Mengmeng (2017), Jiao et al. (2013), Al-Nassan and Fujino (2018), Liu et al. (2019), Ono et al. (2020), Frick et al. (2008), Witteveen et al. (2016), Pierre et al. (2023), Nakanishi et al. (2022), Owen et al. (2019), Preau et al. (2019), Hill et al. (2017), Witteveen et al. (2019), Bloise et al. (2016)
Disuse model	Tail or hind limb suspension/Limb immobilisation	4	Easy to administer with fewer side effects	Single inducer and time consuming	Kutz et al. (2023), Yang et al. (2017), Onda et al. (2016), Aihara et al. (2017)
ICU model	MV + Sedation	9 (28.1)	Able to simulate clinical scenarios well	Mortality and costs are high, difficult to operate	Mrozek et al. (2012), Hongli et al. (2012), Matecki et al. (2017), Gayan-Ramirez et al. (2003), Radell et al. (2004), Radell et al. (2002), Tang et al. (2013), Shanely et al. (2002), Zambelli et al. (2019)
	Deep anaesthesia + MV + Pharmacological paralysis	7 (21.9)			Llano-Diez et al. (2016), Addinsall et al. (2022), Corpeno et al. (2014), Llano-Diez et al. (2011), Akkad et al. (2014), Renaud et al. (2013), Ochala et al. (2011c)
	MV + Immobilisation	1 (3.1)			Banduseela et al. (2009)
	MV + NMBA	1 (3.1)			Shimatani et al. (2019)
	Sedation + MV + sepsis + NMBA + CS	4 (12.5)			Banduseela et al. (2013), Aare et al. (2011), Ochala et al. (2011a), Ochala et al. (2011b)
	Sepsis + MV	3 (9.3)			Ackermann et al. (2014), Li et al. (2021), Le Dinh et al. (2018)
	Sepsis + immobilisation	5 (15.6)	Can mimic ICU clinical practice to some extent	Easy to implement compared to other ICU models	Zhang et al. (2019), Habr et al. (2020), Jiang et al. (2022), Laitano et al. (2021), Grunow et al. (2023)
	Denervation + hyperglycaemia	2 (6.2)	—	—	Yamada et al. (2018), Barnes et al. (2015)
Other models	Others: Pancreatitis + abdominal hypertension/Induced ARDS/Burned model/Hyperglycaemia model	5	—	Infrequently used, with less supporting literature	Liao et al. (2018), Radigan et al. (2019), Wu et al. (2010), Ono et al. (2022), Callahan and Supinski (2014)

ARDS: acute respiratory distress syndrome; CLP: caecal ligation perforation; CS: corticosteroids; IP: intraperitoneal injection; IV: intravenous injection; IN: intranasal inoculation; LPS: lipopolysaccharide; NMBA: neuromuscular blocking agent;/: none; N: the number of outcomes.

### Outcomes

The types of target muscles among the included studies are summarised in Table 1. Significant variations were observed in the intervention duration and time of outcome assessment among different models. For the sepsis model, sampling time ranged from 6 h to 61 days, with the most common occurring within 24 h 46.5% (n = 20/43). ICU models were typically sampled at 4 hours–4 days (68.8%, n = 22/32), 5–8 days (62.5%, n = 20/32) and 9–14 days (28.1%, n = 9/32) of post-intervention. Diaphragm muscle samples were typically collected within 24 h (69.2%, n = 18/26). For limb muscles, sampling occurred at various intervals: 6 hours–2 days (46.9%, n = 30/64), 3–4 days (32.8%, n = 21/64), 5–7 days (42.2%, n = 27/64) and more than 8 days (35.9%, n = 23/64) after induction. Notably, only 42.9% of the studies (n = 36/84) reported outcomes at more than two time points. Further details are provided in Supplementary Table S2.

Skeletal muscle weakness and atrophy are key features of ICU-AW (Wang et al., 2020). Muscle weakness was described in 27.4% (n = 23/84) of the studies, whereas muscle atrophy was noted in 25.0% (n = 21/84). Meanwhile, 39.3% of the studies (n = 33/84) focused on both muscle function decreasing and atrophy, with only 7 studies not demonstrating pathological changes in the ICU-AW model group. In addition, 42.9% of the studies (n = 36/84) evaluated the level of atrophy markers, and 20.2% (n = 17/84) reported changes in actin and/or myosin protein levels.

Sepsis animal models replicated muscle weakness in 27.9% of the studies, muscle atrophy in 23.3% and both in 39.5%. Four studies did not specify the presence of muscle weakness or atrophy. Only 9.3% reported changes in myosin and actin but without priority myosin lost. By contrast, ICU models induced by MV with other factors showed muscle weakness in 28.1% of the studies, muscle atrophy in 25.0% and both in 37.5%. Additionally, 40.6% of these studies indicated changes in myosin and actin proteins, with some showing a significant decrease in the myosin/actin ratio. More than a fifth (21.9%) of the studies reported changes in type I and/or type II muscle fibres, with 5 studies demonstrating significant damage to type II muscle fibres. The sepsis immobilisation model showed muscle weakness and atrophy in 40.0% of the studies and both features in 20%, with some studies reporting preferential myosin loss and a decrease in type II muscle fibers. For details, see Supplementary Table S3.

Muscular pathological changes were reported in 83.3% of the studies (n = 70/84), including muscle fibre morphology, cross-sectional muscle area (CSA), fibre diameters, myosin and/or actin protein, atrophic genes or proteins, and muscle type. The imaging methods utilised in the included studies were mostly HE and electron microscopy. A total 57.1% of the studies (n = 48/84) observed the changes via electrophysiological or electromyographic examinations of muscles, such as excitability, CMAP and muscular maximal twitch and tetanic force. Animal weight change was monitored in 48.9% of the studies (n = 41/84), while changes in muscle weight were described in 39.3% (n = 33/84). Daily behavioural changes in animals were assessed in 11.9% of the studies (n = 10/84), and animal grip strength was measured in 16.7% (n = 14/84). Further details are provided in Table 3.

**TABLE 3:** Details of outcome measurement.

Model type	Indicators		N	Limb muscle	Diaphragm
Sepsis model	Muscle functions	Electrophysiological examination	23	Nardelli et al. (2016), Goossens et al. (2021), Alamdari et al. (2012), Weckx et al. (2022), Crowell and Lang (2021), Rocheteau et al. (2015), Vankrunkelsven et al. (2022), Wang and Wu (2020), Li et al. (2023), Nardelli et al. (2013), Cankayali et al. (2007), Rossignol et al. (2008), Liu et al. (2014), Vankrunkelsven et al. (2023), Frick et al. (2008), Witteveen et al. (2019)	Zhang et al. (2016), Jude et al. (2020), Supinski et al. (2020a), Supinski et al. (2014), Supinski et al. (2020b), Mengmeng (2017), Jiao et al. (2013), Witteveen et al. (2019)
		Grip strength	10	Zheng et al. (2023), Rocheteau et al. (2015), Li et al. (2023), Chen et al. (2023), Wang et al. (2023), Ono et al. (2020), Witteveen et al. (2016), Nakanishi et al. (2022), Hill et al. (2017), Witteveen et al. (2019)	—
	Muscle atrophy	Body weight	22	Liu et al. (2023), Zheng et al. (2023), Schmitt et al. (2023), Crowell and Lang (2021), Vankrunkelsven et al. (2022), Wang and Wu (2020)0, Hou et al. (2023), Chen et al. (2023), Wang et al. (2023), Rossignol et al. (2008), Vankrunkelsven et al. (2023), Al-Nassan and Fujino (2018), Ono et al. (2020), Frick et al. (2008), Witteveen et al. (2016), Pierre et al. (2023), Nakanishi et al. (2022), Owen et al. (2019), Preau et al. (2019), Hill et al. (2017), Witteveen et al. (2019)	Jude et al. (2020)
		Muscle mass	19	Zheng et al. (2023), Weckx et al. (2022), Schmitt et al. (2023), Crowell and Lang (2021), Hou et al. (2023), Hou et al. (2021), Chen et al. (2023), Hahn et al. (2020), Wang et al. (2023), Liu et al. (2014), Vankrunkelsven et al. (2023), Al-Nassan and Fujino (2018), Ono et al. (2020) Frick et al. (2008), Owen et al. (2019), Preau et al. (2019), Hill et al. (2017)	Jude et al. (2020), Cankayali et al. (2007)
		Morphological changes in muscle (CSA; Myofibre morphology; fibre type, HE)	25	Goossens et al. (2021), Alamdari et al. (2012), Liu et al. (2023), Zheng et al. (2023), Schmitt et al. (2023), Rocheteau et al. (2015), Vankrunkelsven et al. (2022), Cao et al. (2021), Wang and Wu (2020), Hou et al. (2021), Li et al. (2023), Hahn et al. (2020), Zanders et al. (2022), Wang et al. (2023), Vankrunkelsven et al. (2023), Al-Nassan and Fujino (2018), Liu et al. (2019), Ono et al. (2020), Pierre et al. (2023), Nakanishi et al. (2022), Owen et al. (2019), Preau et al. (2019), Bloise et al. (2016)	Jude et al. (2020), Liu et al. (2014), Bloise et al. (2016)
		Expression of atrophic genes or proteins	18	Jude et al. (2020), Liu et al. (2023), Zheng et al. (2023), Weckx et al. (2022), Vankrunkelsven et al. (2022), Cao et al. (2021), Hou et al. (2023), Li et al. (2023), Chen et al. (2023), Hahn et al. (2020), Zanders et al. (2022), Wang et al. (2023), Vankrunkelsven et al. (2023), Al-Nassan and Fujino (2018), Ono et al. (2020), Pierre et al. (2023), Preau et al. (2019)	Supinski et al. (2014)
		Sarcomeric proteins Myosin and/or actin content, MHC	11	Goossens et al. (2021), Crowell and Lang (2021), Vankrunkelsven et al. (2022), Hahn et al. (2020), Zanders et al. (2022), Ono et al. (2020), Witteveen et al. (2016), Witteveen et al. (2019), Bloise et al. (2016)	Supinski et al. (2014), Supinski et al. (2020b), Witteveen et al. (2019), Bloise et al. (2016)
	Behavioural indicators	Daily behaviour, activities	8	Jude et al. (2020), Liu et al. (2014), Ono et al. (2020), Frick et al. (2008), Owen et al. (2019), Preau et al. (2019)	Zhang et al. (2016), Vankrunkelsven et al. (2023)
Disuse model	Muscle Functions	Electrophysiological examination	1	Yang et al. (2017)	—
		Body weight	2	Onda et al. (2016), Aihara et al. (2017)	—
		Muscle mass	4	Kutz et al. (2023), Yang et al. (2017), Onda et al. (2016), Aihara et al. (2017)	—
	Muscle atrophy	Morphological changes in muscle (CSA, fibre type, HE)	2	Yang et al. (2017), Aihara et al. (2017)	—
		Expression of atrophic genes or proteins	1	Onda et al. (2016)	—
		Daily behaviour, activities	1	Onda et al. (2016)	
ICU model	Muscle functions	Electrophysiological examination	21	Mrozek et al. (2012), Gayan-Ramirez et al. (2003), Addinsall et al. (2022), Corpeno et al. (2014), Llano-Diez et al. (2011), Renaud et al. (2013), Ochala et al. (2011c), Banduseela et al. (2009), Aare et al. (2011), Ochala et al. (2011a), Ackermann et al. (2014), Habr et al. (2020), Laitano et al. (2021), Yamada et al. (2018)	Mrozek et al. (2012), Matecki et al. (2017), Gayan-Ramirez et al. (2003), Radell et al. (2002), Zambelli et al. (2019), Llano-Diez et al. (2016), Llano-Diez et al. (2011), Ochala et al. (2011b), Li et al. (2021), Le Dinh et al. (2018)
		Grip strength	2	Habr et al. (2020), Jiang et al. (2022)	—
	Muscle atrophy	Body weight	13	Mrozek et al. (2012), Gayan-Ramirez et al. (2003), Shanely et al. (2002), Addinsall et al. (2022), Corpeno et al. (2014), Llano-Diez et al. (2011), Renaud et al. (2013), Zhang et al. (2019), Habr et al. (2020), Laitano et al. (2021), Grunow et al. (2023), Yamada et al. (2018)	Mrozek et al. (2012), Hongli et al. (2012), Gayan-Ramirez et al. (2003), Shanely et al. (2002), Llano-Diez et al. (2011), Zhang et al. (2019)
		Muscle mass	17	Gayan-Ramirez et al. (2003), Tang et al. (2013), Shanely et al. (2002), Corpeno et al. (2014), Llano-Diez et al. (2011), Renaud et al. (2013), Habr et al. (2020), Jiang et al. (2022), Laitano et al. (2021), Grunow et al. (2023), Yamada et al. (2018), Barnes et al. (2015)	Gayan-Ramirez et al. (2003), Tang et al. (2013), Llano-Diez et al. (2011)
		Morphological changes in muscle (CSA, fibre type, HE)	24	Gayan-Ramirez et al. (2003), Tang et al. (2013), Shanely et al. (2002), Corpeno et al. (2014), Llano-Diez et al. (2011), Renaud et al. (2013), Banduseela et al. (2009), Aare et al. (2011), Ochala et al. (2011a), Zhang et al. (2019), Habr et al. (2020), Jiang et al. (2022), Laitano et al. (2021), Grunow et al. (2023), Yamada et al. (2018), Barnes et al. (2015)	Hongli et al. (2012), Gayan-Ramirez et al. (2003), Radell et al. (2004), Tang et al. (2013), Shanely et al. (2002), Zambelli et al. (2019), Llano-Diez et al. (2016)6, Llano-Diez et al. (2011), Shimatani et al. (2019), Ochala et al. (2011b), Li et al. (2021), Le Dinh et al. (2018), Zhang et al. (2019)
		Expression of atrophic genes or proteins	15	Tang et al. (2013), Corpeno et al. (2014), Llano-Diez et al. (2011), Akkad et al. (2014), Renaud et al. (2013), Ochala et al. (2011c), Banduseela et al. (2009), Aare et al. (2011), Ochala et al. (2011a), Zhang et al. (2019), Yamada et al. (2018)	Tang et al. (2013), Zambelli et al. (2019), Llano-Diez et al. (2016), Llano-Diez et al. (2011), Ochala et al. (2011b), Li et al. (2021), Zhang et al. (2019)
		Myosin and/or actin content	12	Corpeno et al. (2014), Llano-Diez et al. (2011), Akkad et al. (2014), Renaud et al. (2013), Ochala et al. (2011c), Banduseela et al. (2009), Banduseela et al. (2013), Aare et al. (2011), Ochala et al. (2011a), Habr et al. (2020), Yamada et al. (2018)	Radell et al. (2004), Llano-Diez et al. (2011)

CSA: cross-sectional area; MV: mechanical ventilation;/: none; MHC: myosin heavy chain; HE: HE, staining; N: the number of outcomes.

High animal mortality rate presents a significant challenge in ICU-AW animal research, with only 42.9% of the studies (n = 36/84) reporting animal mortality rates. Notably, the sepsis model predominantly reported mortality within 48 h (25.6%, n = 11/43), with a mean mortality rate of 35.3%. In the ICU model, mortality was reported in only 21.9% of the studies (n = 7/32), predominantly within 24 h, with a mean mortality rate of 24.6%. Only 71.4% of the studies (n = 60/84) utilised various life support treatments, including antibiotics, analgesia, fluid resuscitation, warmth maintenance, nutritional infusion and vital signs monitoring. Analgesia (n = 23), antibiotics (n = 23) and fluid resuscitation (n = 38) were the top three life support strategies used among the included studies.

## Discussion

Animal experiments offer advantages in understanding the pathophysiological mechanisms of muscle dysfunction during critical illness and exploring intervention targets due to their reproducibility and environmental control. This is the first scoping review providing a comprehensive overview of current animal models of ICU-AW. It is based on a systematic retrieval and inclusion of all potential eligible studies, ensuring a rigorous and systematic approach to data analysis and synthesis, which offers a clear advantage over narrative reviews. The key findings are as follows: (i) There are currently no standard ICU-AW animal model. ICU animal models, which are commonly induced by MV and sepsis with other factors, were the most effective methods for inducing ICU-AW. (ii) The mortality rates of ICU models averaged 24.6%, though not all models reported mortality rates. (iii) The assessment and reporting of ICU-AW outcomes were often inadequate. (iv) Important risk factors, such as being elderly and female, were frequently overlooked, and issues related to trial design and sample estimation were inadequately addressed.

An ideal animal model for ICU-AW should be simple, cost-effective and capable of replicating its pathophysiological changes and risk factors (Zou et al., 2018). However, there are currently no standard ICU-AW animal models fully replicating the multiple risk factors and interventions present in ICU clinical settings, thus posing significant challenges for animal models (Yang et al., 2018). In our present review, ICU models, particularly those involving MV with various ICU-AW risk factors, are the most effective methods for inducing ICU-AW. In particular, we found that ICU models induced by MV with other risk factors showed muscle weakness and atrophy as well as a significant decrease in the myosin/actin ratio and type II muscle fibres, mimicking the pathological characteristic in human ICU-AW (Friedrich et al., 2015). However, implementing such models is challenging.

For example, rats and porcine induced with MV after deep anaesthesia or sedation, along with life support and other treatments, can effectively replicate the muscle wasting and weakness observed in humans with ICU-AW (Addinsall et al., 2022; Corpeno et al., 2014; Llano-Diez et al., 2011). However, the need for continuous 24-h monitoring and therapeutic interventions limits their practical use due to cost and labour demands (Lad et al., 2020). Ventilation durations also very significantly, lasting 18 h in mice (Tang et al., 2013), 5–8 days or 9–14 days in rats (Llano-Diez et al., 2011; Akkad et al., 2014; Renaud et al., 2013) and 5 days in pigs (Radell et al., 2004; Banduseela et al., 2013; Ochala et al., 2011a). While advancements in equipment miniaturisation have addressed many technical obstacles in establishing ICU procedures for rat or murine experiments (Kim, 2021), challenges remain, including the complexity of model development and management, high time and financial costs, high mortality rates and diagnostic difficulties. The limited applicability of heavy sedation and neuromuscular blocking agents, used in only a subset of ICU patients, also hinders the complete replication of the ICU clinical environment in these animal models (Zhu et al., 2019).

Additionally, the sepsis immobilisation model exhibits selective myosin filament loss, particularly in type II fibres, which aligns with ICU-AW pathology (Friedrich et al., 2015). Immobilised septic patients without MV actually represent a significant proportion of ICU patients. Animal models combining sepsis with limb immobilisation, also called sepsis-acquired muscle weakness (Latronico et al., 2023), have gained popularity due to their simplicity and ability to induce severe muscle weakness and atrophy (Ochala et al., 2011a). Thus, the sepsis with immobilisation model, coupled with appropriate resuscitation measures, offers a simpler and reliable approach for inducing muscle weakness and atrophy in critical illness animal models. It is especially suitable for laboratories lacking sufficient resources.

Although sepsis and disuse models were also used to explore ICU-AW in our review, none of the studies utilising these models found the typical pathological changes of ICU-AW ‘preferential myosin proteolysis’. Four studies on sepsis models reported decreases in myosin and actin content without preferential myosin proteolysis (Goossens et al., 2021; Witteveen et al., 2019). These findings support that sepsis-induced myopathy differs from the pathology of ICU-AW and cannot replicate ICU-AW accurately (Friedrich et al., 2015) and that the sepsis model is not recommended for inducing ICU-AW. Therefore, a standardised ICU-AW model must be developed in the near future to enhance reproducibility.

Our review revealed that the assessment and reporting of ICU-AW outcomes in studies were inadequate. It is crucial to evaluate whether animal models replicate the changes associated with ICU-AW to assess the validity of the models. Handgrip strength, electromyography or muscle biopsy is the primary diagnostic modality for identifying ICU-AW in patients (Latronico et al., 2023). While muscle atrophy is consistently assessed, about half of the studies involved electrophysiological parameters or grip strength. Specifically, 42.9% analysed the expression of atrophic genes or proteins, 20.2% discussed changes in myosin or actin and only 39.3% reported both weakness and atrophy. None of the studies evaluated if they could fully replicate the weakness and atrophy characteristics of CIP or CIM or distinguish the subtype of ICU-AW. Therefore, future research should adequately assess both muscle weakness and atrophy outcomes to distinguish the subtypes of ICU-AW.

The duration of intervention and timing of outcome assessment significantly influence the results, with considerable heterogeneity among the included studies (Ochala et al., 2011b). Only 42.9% reported outcomes at more than two time points. Diaphragm muscle samples were typically collected within 24 h, while limb muscle sampling varied from 6 h to 61 days post induction. Diaphragm fibre atrophy occurred after 12 h of MV and worsened with prolonged ventilation, particularly within the first 72 h (Schepens et al., 2015). Critically ill patients experienced a significant loss of nearly 2% of skeletal muscle per day during the first week of ICU admission (Latronico et al., 2023), with non-excitable muscle membranes observed in response to direct muscle stimulation (Bierbrauer et al., 2012). A complete sarcomere disruption was documented in 100% of patients with CIM 7 days after ICU discharges (Dos Santos et al., 2016). Variations in mortality rates and the timing of animal execution also affected outcome observations over time. In particular, ICU-AW often occurred 7 days after the onset of critical illness (Fazzini et al., 2023), and early deaths hindered the observation of muscle changes throughout the study period. Therefore, assessing muscle function and atrophy at different time points is essential for understanding the temporal contribution of ICU-AW development.

Female and older critically ill patients were found to be more susceptible to developing ICU-AW (Yang et al., 2023; Wu et al., 2022). Recent studies indicated that women experience muscle loss rates approximately 2.6–3.4 times higher than men (Wu et al., 2022), particularly in septic ICU patients (Zaragoza-García et al., 2023). This gender disparity is associated with lower insulin sensitivity index and myocyte cross-sectional area (MCSA) of type IIa fibres in critically ill female patients (Engelhardt et al., 2022). Moreover, testosterone replacement therapy has shown potential in improving grip strength in patients with ICU-AW (Samuel and Swee, 2023). Interestingly, most included studies predominantly utilised male animals, while some failed to specify the animal gender. In addition, nearly half of the included studies did not report the animal age, with only 29.8% utilising adult animals and 2 studies involving older animals. This discrepancy underscores the importance of clearly defining the characteristics of animals in future research. Furthermore, prioritising the use of adult or older animals can better align experimental conditions with clinical observations in ICU patients.

Transparent reporting of research methods and findings, along with rigorous research design, is crucial for ensuring reproducibility (Percie du Sert et al., 2020). However, several quality issues were identified in the included preclinical studies. (i) Approximately 50% of the studies performed randomisation, but most RCT studies lacked clarity in their randomisation methods. (ii) None of the included studies reported using blinding, 27.4% of the studies failed to report the sample size, and none clarified whether the group sizes were similar at the start. Only one study estimated the sample size. (iii) The number of animals was unequal between the intervention and control groups. Mortality rate may account for the unequal group sizes. (iv) Essential information to assess risk of bias, such as allocation method, concealment methods and animal housing measures (e.g., environment, housing conditions, feeding and watering, health monitoring, handling and care), was rarely reported. These findings are consistent with previous studies (Percie du Sert et al., 2020; Guo et al., 2023). Although researchers may not always know whether mice or rats are from the same litter, after the induction of ICU-AW, it is expected that these animals will be housed individually. No study has addressed the clustering effects caused by hierarchies in the design and analysis, where measurements from the same cage or litter or cells from the same animal show greater similarity (Percie du Sert et al., 2020). Addressing such relationships in statistical analyses by incorporating or aggregating outcome variables to the cage, litter or animal level is crucial. These quality issues should be urgently considered to ensure accurate conclusions and guide future research effectively.

Our scoping review identified several shortcomings in previous animal studies of ICU-AW, including a lack of blinding, sample size estimation and randomisation, as well as limited reporting on animal characteristics, intervention and mortality, with a focus predominantly on male and young animals. To tackle these issues and improve future research, we recommend the following: (i) Standardized ICU-AW animal models that are easier to implement should be developed. Ensure that clinical scenarios are accurately replicated, establish clear criteria for successful model establishment, and create corresponding research design guidelines to enhance reproducibility. (ii) Scientific rigor in study design and transparent reporting should be improved using the 21 checklist items outlined in ARRIVE guidelines 2.0 (Percie du Sert et al., 2020) to increase reproducibility. (iii) Adult or elderly male and female animals should be considered to better reflect the clinical characteristics of ICU patients.

However, our scoping review have several limitations. Firstly, risk of bias assessments were not conducted due to the methodology of scoping reviews. This may lead to include some low-quality studies, potentionally affecting the robustness of the findings on ICU-AW animal models. Secondly, variations in intervention measures, animal species, study designs, and outcome measures contribute to substantial heterogeneity, which only allows for descriptive analysis rather than meta-analysis. Additionally, the data extraction process based on predefined categories may have oversimplified the information presented in the studies, potentially overlooking some valuable details.

## Conclusion

In this scoping review, we outlined the current preclinical models of ICU-AW. While many preclinical models are used to study ICU-AW, the reporting of methodological details is often incomplete, compounding the limitations of the preclinical studies identified in this review. Although current ICU animal models can mimic the clinical situation and pathological changes of human ICU-AW patients, there is no standard model. MV animal models incorporating other ICU condition factors can simulate complex clinical scenarios well, but they are challenging to implement and manage. The sepsis immobilisation model offers a simpler alternative, especially for laboratories lacking sufficient resources. However, sepsis and disuse models cannot mimic the pathological characteristics of ICU-AW and are not recommended for inducing ICU-AW. Future preclinical studies aim to design a standard ICU-AW model that can enhance reproducibility and improve scientific rigour in identifying the mechanisms underlying ICU-AW.

## Data Availability

The original contributions presented in the study are included in the article/Supplementary Material, further inquiries can be directed to the corresponding authors.
