# Supplementary material for: A scoping review of preclinical intensive care unit-acquired weakness models
Source: Front Physiol. 2024 Oct 2;15:1423567. doi: 10.3389/fphys.2024.1423567 (PMC11480018; doi:10.3389/fphys.2024.1423567)
Supplement: Supplementary file 1 [file Table1.docx]

**Table 1 Search** **strategies in every database**

| China national knowledge infrastructure (CNKI) | | |
| --- | --- | --- |
| 01/02/2024 | | |
| #1 | SU=('ICU'+'重症监护室'+'重症监护病房'+'脓毒症'+'脓毒症休克'+'脓毒血症'+'危重症'+'严重创伤'+'多器官功能衰竭'+'制动'+'废用'+'高血糖'+'多发伤'+'严重烧伤'+'悬吊'+'LPS'+'脂多糖'+'CLP'+'ARDS'+'类固醇'+'糖皮质激素'+'盲肠结扎穿孔') | 892,900 |
| #2 | SU=('ICU-AW' +'肌无力' +'肌麻痹'+'肌萎缩'+'ICU获得性衰弱'+'ICU获得性肌无力'+'ICU获得性肌肉萎缩'+'危重病肌病'+'危重病性多神经肌病'+'危重病性多发性神经病'+'CIM'+'CIP'+'CIPNM'+'肌肉衰弱'+'危重症神经病'+' 危重症多神经肌病'+'脓毒症相关性肌无力'+'脓毒症相关性衰弱'+'脓毒症肌病'+'膈肌萎缩'+'呼吸肌萎缩'+'呼吸肌无力'+'膈肌无力') | 84,300 |
| #3 | SU=('小鼠'+'大鼠'+'豚鼠'+'兔'+'狗'+'犬'+'动物'+'荷兰猪') | 3,600,500 |
| #4 | #1 AND #2 AND #3 | 527 |
| Wanfang database | | |
| 01/02/2024 | | |
| #1 | 主题:(ICU OR 重症监护室 OR 重症监护病房 OR 脓毒症 OR 脓毒症休克 OR 脓毒血症 OR 危重症 OR 严重创伤 OR 多器官功能衰竭 OR 制动 OR 废用 OR 高血糖 OR 多发伤 OR 严重烧伤 OR 悬吊 OR LPS OR 盲肠结扎穿孔 OR 脂多糖 OR CLP OR ARDS OR 类固醇 OR 糖皮质激素) | 2,459,924 |
| #2 | 主题:(ICU-AW OR 肌无力 OR 肌麻痹 OR 肌萎缩 OR ICU获得性衰弱 OR ICU获得性肌无力 OR ICU获得性肌肉萎缩 OR 危重病肌病 OR 危重病性多神经肌病 OR 危重病性多发性神经病 OR CIM OR CIP OR CIPNM OR 肌肉衰弱 OR 危重症神经病 OR 危重症多神经肌病 OR 脓毒症相关性肌无力 OR 脓毒症相关性衰弱 OR 脓毒症肌病 OR 膈肌萎缩 OR 呼吸肌萎缩 OR 呼吸肌无力 OR 膈肌无力) | 70,530 |
| #3 | 主题:(小鼠 OR 大鼠 OR 豚鼠 OR 兔 OR 狗 OR 犬 OR 动物 OR 荷兰猪) | 2,050,750 |
| #4 | #1 AND #2 AND #3 | 698 |
| Sinomed (CBM) | | |
| 01/02/2024 | | |
| #1 | ICU OR 重症监护室 OR 重症监护病房 OR 脓毒症 OR 脓毒症休克 OR 脓毒血症 OR 危重症 OR 严重创伤 OR 多器官功能衰竭 OR 制动 OR 废用 OR 高血糖 OR 多发伤 OR 严重烧伤 OR 悬吊 OR LPS OR 盲肠结扎穿孔 OR 脂多糖 OR CLP OR ARDS OR 类固醇 OR 糖皮质激素 | 349,020 |
| #2 | ICU-AW OR 肌无力 OR 肌麻痹 OR 肌萎缩 OR ICU获得性衰弱 OR ICU获得性肌无力 OR ICU获得性肌肉萎缩 OR 危重病肌病 OR 危重病性多神经肌病 OR 危重病性多发性神经病 OR CIM OR CIP OR CIPNM OR 肌肉衰弱 OR 危重症神经病 OR 危重症多神经肌病 OR 脓毒症相关性肌无力 OR 脓毒症相关性衰弱 OR 脓毒症肌病 OR 膈肌萎缩 OR 呼吸肌萎缩 OR 呼吸肌无力 OR 膈肌无力 | 31,237 |
| #3 | 小鼠 OR 大鼠 OR 豚鼠 OR 兔 OR 狗 OR 犬 OR 动物 OR 荷兰猪 | 844,281 |
| #4 | #1 AND #2 AND #3 | 231 |
| VIP databases | | |
| 01/02/2024 | | |
| #1 | M=(ICU OR 重症监护室 OR 重症监护病房 OR 脓毒症 OR 脓毒症休克 OR 脓毒血症 OR 危重症 OR 严重创伤 OR 多器官功能衰竭 OR 制动 OR 废用 OR 高血糖 OR 多发伤 OR 严重烧伤 OR 悬吊 OR LPS OR 盲肠结扎穿孔 OR 脂多糖 OR CLP OR ARDS OR 类固醇 OR 糖皮质激素) | 193,933 |
| #2 | M=(ICU-AW OR 肌无力 OR 肌麻痹 OR 肌萎缩 OR ICU获得性衰弱 OR ICU获得性肌无力 OR ICU获得性肌肉萎缩 OR 危重病肌病 OR 危重病性多神经肌病 OR 危重病性多发性神经病 OR CIM OR CIP OR CIPNM OR 肌肉衰弱 OR 危重症神经病 OR 危重症多神经肌病 OR 脓毒症相关性肌无力 OR 脓毒症相关性衰弱 OR 脓毒症肌病 OR 膈肌萎缩 OR 呼吸肌萎缩 OR 呼吸肌无力 OR 膈肌无力) | 45,366 |
| #3 | M=(小鼠 OR 大鼠 OR 豚鼠 OR 兔 OR 狗 OR 犬 OR 动物 OR 荷兰猪） | 695,790 |
| #4 | #1 AND #2 AND #3 | 65 |
| Pubmed | | |
| 01/02/2024 | | |
| #1 | ICU[Text Word] OR intensive care unit[Text Word] OR critically ill[Text Word] OR critically illness[Text Word] OR sepsis[Text Word] OR septicopyemia[Text Word] OR septic shock[Text Word] OR severe truma[Text Word] OR severe burns[Text Word] OR multiple injury[Text Word] OR multiple truma[Text Word] OR multiple Organ dysfunction syndrome[Text Word] OR MODS[Text Word] OR unloading[Text Word] OR disuse[Text Word] OR immobilization[Text Word] OR hyperglycaemia[Text Word] OR coli septic peritonitis[Text Word] OR mechanical ventilation[Text Word] OR LPS[Text Word] OR ARDS[Text Word] OR steroid-induced[Text Word] OR CLP[Text Word] | 466,459 |
| #2 | ICU-AW[Text Word] OR amyosthenia[Text Word] OR myoparalysis[Text Word] OR myopath*[Text Word] OR muscle wasting[Text Word] OR muscle weakness[Text Word] OR intensive care unit-acquired weakness[Text Word] OR ICU-acquired weakness[Text Word] OR atrophy[Text Word] OR critical illness polyneuropathy[Text Word] OR CIP[Text Word] OR critical illness myopathy[Text Word] OR CIM[Text Word] OR critical illness polyneuromyopathy[Text Word] OR CIPNM[Text Word] OR disuse[Text Word] OR muscle atrophy[Text Word] OR septic myopathy[Text Word] OR septic weakness[Text Word] OR diaphragm weakness[Text Word] OR SAW[Text Word] OR sepsis acquired weakness[Text Word] OR sepsis related weakness[Text Word] | 159,665 |
| #3 | mouse model[Text Word] OR mice model[Text Word] OR rat model[Text Word] OR guinea pig[Text Word] OR rabbit[Text Word] OR dog[Text Word] OR animal model[Text Word] | 403,302 |
| #4 | #1 AND #2 AND #3 | 403 |
| Embase | | |
| 01/02/2024 | | |
| #1 | 'icu' OR 'intensive care unit' OR 'critically ill' OR 'critically illness' OR 'sepsis' OR 'septicopyemia' OR 'septic shock' OR 'severe truma' OR 'severe burns' OR 'multiple injury' OR 'multiple truma' OR 'multiple organ dysfunction syndrome' OR 'mods' OR 'disuse' OR 'immobilization' OR 'hyperglycaemia' OR 'coli septic peritonitis' OR 'mechanical ventilation' OR 'lps' OR 'ards' OR 'clp' OR 'steroid-induced' | [670,869](http://www.embase.zd.hggfdd.top/) |
| #2 | 'icu-aw' OR 'myoparalysis' OR 'myopath*' OR 'muscle wasting' OR 'muscle weakness' OR 'intensive care unit-acquired weakness' OR 'icu-acquired weakness' OR 'atrophy' OR 'critical illness' OR 'polyneuropathy' OR 'cip' OR 'critical illness myopathy' OR 'cim' OR 'critical illness polyneuromyopathy' OR 'cipnm' OR 'disuse' OR 'muscle atrophy' OR 'septic myopathy' OR 'septic weakness' OR 'diaphragm weakness' OR 'saw' OR 'sepsis acquired weakness' OR 'sepsis related weakness' | [264,144](http://www.embase.zd.hggfdd.top/) |
| #3 | 'mouse model' OR 'mice model' OR 'rat model' OR 'guinepig' OR 'rabbit' OR 'dog' OR 'animal model' | [1,338,473](http://www.embase.zd.hggfdd.top/) |
| #4 | #1 AND #2 AND #3 | 1,601 |
| Ovide Medline | | |
| 01/02/2024 | | |
| #1 | (ICU OR intensive care unit OR critically ill OR critically illness OR sepsis OR septicopyemia OR septic shock OR severe truma OR severe burns OR multiple injury OR multiple truma OR multiple organ dysfunction syndrome OR MODS OR unloading OR disuse OR immobilization OR hyperglycaemia OR coli septic peritonitis OR mechanical ventilation OR LPS OR ARDS OR CLP OR steroid-induced).tw | 446,311 |
| #2 | (ICU-AW OR amyosthenia OR myoparalysis OR myopath* OR muscle wasting OR muscle weakness OR intensive care unit-acquired weakness OR ICU-acquired weakness OR atrophy OR critical illness polyneuropathy OR CIP OR critical illness myopathy OR CIM OR critical illness polyneuromyopathy OR CIPNM OR disuse OR muscle atrophy OR septic myopathy OR septic weakness OR diaphragm weakness OR SAW OR sepsis acquired weakness OR sepsis related weakness).tw. | 142,803 |
| #3 | (mouse model OR mice model OR rat model OR guinea pig OR rabbit OR dog OR animal model).tw. | 364,716 |
| #4 | #1 AND #2 AND #3 | 376 |
| Conchrone | | |
| 01/02/2024 | | |
| #1 | "ICU" OR "intensive care unit" OR "critically ill" OR "critically illness" OR sepsis OR septicopyemia OR "septic shock" OR "severe truma" OR "severe burns" OR "multiple injury" OR "multiple truma" OR "multiple organ dysfunction syndrome" OR "MODS" OR "unloading" OR "disuse" OR "immobilization" OR "hyperglycaemia" OR "coli septic peritonitis" OR "mechanical ventilation" OR "LPS" OR "ARDS" OR "steroid-induced" OR "CLP" | 60,664 |
| #2 | "ICU-AW" OR "amyosthenia" OR "myoparalysis" OR myopath* OR "muscle wasting" OR "muscle weakness" OR "intensive care unit-acquired weakness" OR "ICU-acquired weakness" OR "atrophy" OR "critical illness polyneuropathy" OR "CIP" OR "critical illness myopathy" OR "CIM" OR "critical illness polyneuromyopathy" OR "CIPNM" OR disuse OR "muscle atrophy" OR "septic myopathy" OR "septic weakness" OR "diaphragm weakness" OR SAW OR "sepsis acquired weakness" OR "sepsis related weakness" | 14,162 |
| #3 | "mouse model" OR "mice model" OR "rat model" OR "guinea pig" OR rabbit OR dog OR "animal model" | 12,075 |
| #4 | #1 AND #2 AND #3 | 25 |
| Web of Science | | |
| 01/02/2024 | | |
| #1 | "ICU" OR "intensive care unit" OR "critically ill" OR "critically illness" OR "sepsis" OR "septicopyemia" OR "septic shock" OR "severe truma" OR "severe burns" OR "multiple injury" OR "multiple truma" OR "multiple organ dysfunction syndrome" OR "MODS" OR "unloading" OR "disuse" OR "immobilization" OR "hyperglycaemia" OR "coli septic peritonitis" OR "mechanical ventilation" OR "LPS" OR "ARDS" OR "steroid-induced" OR "CLP" | 559,877 |
| #2 | "ICU-AW" OR "amyosthenia" OR "myoparalysis" OR "myopath*" OR "muscle wasting" OR "muscle weakness" OR "intensive care unit-acquired weakness" OR "ICU-acquired weakness" OR atrophy OR "critical illness polyneuropathy" OR CIP OR "critical illness myopathy" OR" CIM" OR "critical illness polyneuromyopathy" OR "CIPNM" OR "disuse" OR "muscle atrophy" OR "septic myopathy" OR "septic weakness" OR "diaphragm weakness" OR "SAW" OR "sepsis acquired weakness" OR "sepsis related weakness" | 210,052 |
| #3 | "mouse model" OR "mice model" OR "rat model" OR "guinea pig" OR "rabbit" OR "dog" OR "animal model" | 542,324 |
| #4 | #1 AND #2 AND #3 | 631 |

**Table 2 Characteristics of included studies**

| **Study** | **Year** | **Country** | **Objective** | **Random** | **Species** | **Gender** | **Age**  **(week)** | **Weight(g)** | **Induction methods** | **Life support techniques** | **Sample size** | **Sample size estimation** | **Muscle Types** | **Muscle Results** | **Points of observation** | **Behavioral indicators** | **Muscle function indicators** | **Muscle pathology indicators** | **Body weight or muscle weight** | **Other indicators** |
| --- | --- | --- | --- | --- | --- | --- | --- | --- | --- | --- | --- | --- | --- | --- | --- | --- | --- | --- | --- | --- |
| Sepsis model | | | | | | | | | | | | | | | | | | | | |
| Nardelli P[1] | 2016 | America |  | / | Rats | M | / | / | CLP | Yes | / | No | Limb muscle | Ⅰ; Ⅲ | 1d, 2d, 3d, 4d, 5d, 2w, 1month | / | Motor neuron excitability; Force production reduced | / | / | / |
| Zhang JY[2] | 2017 | China |  | Yes | SD rats | M | / | 215-  240 | CLP | No | Sham(n=12); CLP-6h(n=12); CLP-12 h: n=12 | No | Diaphragm | Ⅲ | 6h, 12h | Daily activities | Neuronal excitability | / | / | / |
| Goossens C [3] | 2021 | Belgium |  | Yes | C57BL/6J mice | M | 24 | / | CLP | Yes | Healthy: n= 47; sepsis: n = 101 | No | Limb muscle | Ⅰ; Ⅱ | 5d | / | Muscle force | Myosin or/and actin protein content; CSA; Sarcomere; Myofibrillar | / | / |
| Jude B[4] | 2020 | French |  | / | Wistar rats | F | / | 203.9±1.2 | CLP | Yes | Con: n=7; Septic: n=7; LY-D0: n=8; LY-D1: n=7 | No | Diaphragm | Ⅰ; Ⅱ | 7d | Food intake | Muscle force; Contractile force (CF) | Muscle mass; Muscle atrophy | Body weight | / |
| Alamdari N[5] | 2012 | America | ④ | / | SD rats | M | / | 50-60 | CLP | No | / | No | Limb muscle | Ⅰ; Ⅱ | 16h | / | Muscle force; Contractility | CSA; Atrophic genes or proteins; CSA | / | / |
| Liu H[6] | 2023 | China |  | Yes | Piglets | M | 9 | / | CLP | Yes | Protocol 1: n = 6 for each: Con, traditional CLP, modified CLP. Protocol 2: n = 6 for each: WT sham Con, WT CLP, ZBED6‐deficient sham Con, and ZBED6‐deficient CLP | No | Limb muscle | Ⅱ | 14d | / | / | CSA; Atrophic genes or proteins; | Body weight; Muscle weight | / |
| Zheng Y[7] | 2023 | China |  | / | C57BL/6J mice | M | 8 to 10 | 22-25 | CLP | Yes | / | No | Limb muscle | Ⅰ; Ⅱ | 1d, 3d, 7d | / | Grip strength | CSA; Atrophic genes or proteins | Body weight; Muscle weight | Inflammatory factor |
| Weckx R[8] | 2023 | Belgium | ④ | Yes | C57BL/6J mice | / | 24 | / | CLP | Yes | / | No | Limb muscle | Ⅰ; Ⅱ | 5d | / | Muscle force | Muscle mass; Atrophic genes or proteins | / | Inflammatory factor |
| Schmitt RE[9] | 2023 | America | ④ | / | C57BL/6J mice | M or F | Adult | / | CLP | Yes | / | No | Limb muscle | Ⅱ | 4d, 8d, 28d | / | / | CSA; Muscle mass | Body weight; Muscle weight | / |
| Crowell KT[10] | 2021 | America |  | Yes | C57BL / 6 mice | M | 10 | 27 | CLP | Yes | / | No | Limb muscle | Ⅰ | 24h, 10d | / | Muscle force | Myosin or/and actin; MHC; Muscle mass | Body weight; Muscle weight | / |
| Rocheteau P[11] | 2015 | French | ④ | / | C57BL/6J mice | M | 6-12 | / | CLP | Yes | / | No | Limb muscle | Ⅰ; Ⅱ | 24h, 48h, 4d, 7d, 21d | / | Muscle regenerative functions; Grip strength | Muscle fiber changes; Stem cell | / | Mitochondrial function; ROS |
| Vankrunkelsven W[12] | 2022 | Belgium | ④ | Yes | C57BL / 6 mice | M | 6 | / | CLP | Yes | / | No | Limb muscle | Ⅰ; Ⅱ | 5d | / | Muscle force | Myofiber morphology; Atrophic genes or proteins; MHC | Body weight | Inflammatory markers |
| Cao YY[13] | 2021 | China |  | / | C57BL/6J mice | M | 8 | 25-28 | CLP | Yes | / | No | Limb muscle | Ⅱ | 72h | / | / | Atrophic genes or proteins; CSA; muscle morphology; Apoptosis. | Muscle weight | / |
| Wang J[14] | 2020 | China | ④ | Yes | SD rats | M | / | / | CLP | Yes | Experimental: n=8; Con: n = 8; Sham: n=8; Con: n = 8 | No | Limb muscle | Ⅰ; Ⅱ | 8d+3w | / | Maximum contractile force; Fatigue index | CSA | Body weight | / |
| Hou YC[15] | 2023 | China | ④ | Yes | C57BL/6J mice | M | 8 | / | CLP | Yes | Con: n = 12;  Sepsis: n=20; SG: n = 20; SL: n = 20; SGL: n = 20 | No | Limb muscle | Ⅱ | 1d, 4d | / | / | Muscle mass; Atrophic genes or proteins; Protein content | Body weight | / |
| Hou YC[16] | 2021 | China | ④ | Yes | C57BL/6J mice | M | 8 | / | CLP | Yes | Con: n = 12; Sepsis: n=20; Glutamine: n=20; Leucine: n=20; Glutamine+ Leucine: n=20 | No | Limb muscle | / | 1d, 4d | / | / | Muscle morphology; Macrophage | Muscle weight | Mitochondrial function; Inflammatory markers |
| Li X[17] | 2023 | China |  | Yes | C57BL/6J mice | M | 8 | / | CLP | Yes | Sham: n=10; CLP-6h: n=10; CLP-12h: n=10; CLP-24h: n=10 | No | Limb muscle | Ⅰ; Ⅱ; Ⅲ | 6h, 12h, 24h | / | CMAP; Grip strength | CSA；Atrophic genes or proteins | / | Mitochondrial function; Inflammatory markers |
| Chen J[18] | 2023 | China | ④ | Yes | C57BL/6J mice | M | 6-8 | 20-25 | CLP or IP. LPS | Yes | / | No | Limb muscle | Ⅰ; Ⅱ | CLP:1d, 4d, 7d LPS:3d, 7d | / | Grip strength | Atrophic genes or proteins; Muscle mass | Body weight; Muscle weight | / |
| Hahn A[19] | 2020 | German |  | / | C57BL/6J mice | M | 18 | / | CLP | No | Sham + vehicle: n = 6; CLP + vehicle: n = 4; Sham + BMS‐345541: n = 6; CLP + BMS‐345531: n = 11 | No | Limb muscle | Ⅱ | 96h | / | / | CSA; Atrophic genes or proteins; MHC；Muscle fiber type | Muscle weight | / |
| Nardelli P[20] | 2013 | America |  | / | Rats | / | / | / | CLP | Yes | / | No | Limb muscle | Ⅲ | 24h | / | Motoneuron excitability | / | / | / |
| Zanders L[21] | 2022 | German |  | / | C57BL/6J mice | M | 20 | / | CLP | No | CLP-AG490: n =15; CLP-solvent: n= 15; Sham-AG490: n=5; Sham-solvent: n = 5 | No | Limb muscle | Ⅱ | 96h | / | / | CSA; Atrophic genes or proteins; Muscle fiber type; MHC | Muscle weight | / |
| Wang C[22] | 2023 | China |  | Yes | C57BL/6J mice | M | 8-12 | 22-26 | CLP | No | / | No | Limb muscle | Ⅰ; Ⅱ | 4d, 8d | / | Grip strength | CSA; Atrophy; Atrophic genes or proteins; Myofiber morphology | Body weight; Muscle weight | Inflammatory markers |
| Supinski GS[23] | 2020 | America | ④ | / | C57BL/6J mice | / | / | / | CLP | Yes | Sham operated Con: n=5-7; CLP: n=5-7; Sham operated animals given taurine: n=5-7; CLP animals given taurine: n=5-7 | No | Diaphragm | Ⅰ | 24h, 48h, 72h | / | Force generating capacity | Proteolytic pathway | / | / |
| Supinski GS[24] | 2014 | America | ④ | / | WT mice | / | / | / | CLP | Yes | n = 9-10 per: WT sham-operated mice, WT CLP-operated mice, CalpOX sham-operated mice, CalpOX CLP-operated mice. | No | Diaphragm | Ⅰ; Ⅱ | 24h | / | Force generating capacity | MHC; Proteolytic pathway; Atrophic genes or proteins; | / | / |
| Cankayali I[25] | 2007 | Turkey |  | Yes | SD rats | M | 2-3 | 200 -250 | CLP | No | Sepsis: n = 20; Sham: n=10 | No | Limb muscle | Ⅰ | 24h | / | CMAP | / | / | / |
| Rossignol B[26] | 2008 | French |  | Yes | Wistar rats | F | 3 | 256 ±10 | CLP | Yes | "Con": n =10; "Sepsis”: n =15; "Sham": n = 8 | No | Limb muscle | Ⅰ | 10d | / | Force generating capacity; Fatigability; Force generation | / | Body weight | / |
| Supinski GS[27] | 2020 | America | ④ | / | CD1 mice | M | / | 25-30 | CLP | Yes | n = 5–6 mice per: Sham-operated animals treated with saline; Cecal ligation puncture (CLP)-operated animals treated with saline; Sham-operated animals treated with SS31; CLP animals treated with SS31 | No | Diaphragm | Ⅰ | 48h | / | Force generating capacity; Fatigability; | Muscle mass; MHC; Proteolytic enzyme activity | / | Mitochondrial function |
| Liu L[28] | 2014 | China | ④ | Yes | SD rats | M | 2–3 | 200–220 | CLP | Yes | Sepsis: Day 1: n=6; Day 3: n=6; Day 7: n=12; Day 14: n=24; Sham sepsis: Day 1: n=6; Day 3: n=6; Day 7: n=6; Day 14: n=6 | No | Limb muscle | Ⅰ | 1d, 3d, 7d, 14d | Spontaneous activities | CMAP | / | Muscle weight | / |
| Vankrunkelsven W[29] | 2023 | Belgium |  | / | C57BL/6J mice | M | 16 | / | CLP | Yes | / | No | Limb muscle | Ⅰ; Ⅱ | 30h, 125h=5d | / | Muscle force | CSA; Atrophic genes or proteins | Body weight; Muscle weight | Mitochondrial function |
| Wang MM[30] | 2017 | China |  | Yes | SD rats | M | / | 250±20 | IP. LPS | No | Con: n=8; Experimental: n=14 | No | Diaphragm | Ⅰ | 24h | Respiratory frequency | Muscle force; Fatigability | Myofiber morphology | / | / |
| Jiao GY[31] | 2013 | China |  | / | Rats | M | Adult | 250 - 350 | IP. LPS | Yes | Received endotoxin: n=8; Received placebo: n=8 | No | Diaphragm | Ⅰ | 24h | / | Muscle force; Fatigability | / | / | Mitochondrial structure |
| Al-Nassan S[32] | 2018 | Jordan |  | Yes | ICR mice | M | Adult | 30~32 | IP. LPS | No | Sports：Exercise+ LPS: n = 6; Exercise+ PBS: n = 6. Sedentary: Con: n = 6; LPS: n = 6 | No | Limb muscle | Ⅱ | 24h | / | / | CSA; Atrophic genes or proteins; Muscle fiber type | Body weight; Muscle weight | / |
| Liu L[33] | 2019 | China |  | / | BALB/c mice | M | 6-8 | 17-25 | IP. LPS | No | 8 per: (1) normal; (2) blank; (3) negative Con; (4) miR-140 mimic; (5) miR-140 inhibitor; (6) siRNA-WNT 11; (7) miR-140 inhibitor siRNA-WNT 11 | No | Limb muscle | Ⅱ | / | / | / | Myofiber morphology; CSA; Cell apoptosis | / | Inflammatory markers |
| [Yuko Ono](https://pubmed.ncbi.nlm.nih.gov/?term=Ono%20Y%5bAuthor%5d)[34] | 2020 | Japan | ④ | / | C57BL/6J mice | M | 8-12 | / | IP. LPS | No | / | No | Limb muscle | Ⅰ; Ⅱ | 2d | Food iintake | Grip strength | CSA; MyHC; Atrophic genes or proteins; Myofiber morphology | Body weight; Muscle weight | / |
| Frick CG[35] | 2008 | German |  | Yes | SD rats | M | / | 342± 33 | IV. E. coli | Yes | E. coli: day 3: n = 20; day 7: n = 25; day 14: n = 22. Saline: day: 3 n = 10; day 7: n = 10; day 14: n = 11 | No | Limb muscle | Ⅰ; Ⅱ | 3d, 7d, 14d | Food iintake | Tetanic tensions; Contraction force | Muscle mass | Body weight | Inflammatory markers |
| Witteveen E[36] | 2016 | Netherland | ④ | Yes | C57BL/6J mice | M | 8, 13 | / | IP. E. coli | Yes | E. coli+ antibiotics: n=30 young, n=30 old; Con+ antibiotics: n=12 young, =12 old; Con: n=12 young n=12 old | No | Limb muscle | / | 12h, 24h, 48h, 72h | / | Grip strength | Myosin or/and actin protein | Body weight | / |
| Pierre A[37] | 2023 | French |  | / | C57BL/6J mice | M | 7 | / | IP. cecal plasma | Yes | Sepsis n = 17; Sham fed (SF) n = 13; Sham pair fed (SPF) n = 12 | No | Limb muscle | Ⅱ | 24h | / | / | CSA; Atrophic genes or proteins; Autophagy | Body weight | Mitochondrial function |
| Nakanishi N[38] | 2022 | Japan |  | / | C57BL/6J mice | M | 7 | / | IP. cecal plasma | No | / | No | Limb muscle | Ⅰ; Ⅱ | 7d, 14d, 20d, 35d, 61d | / | Grip strength | CSA | Body weight | / |
| Owen AM[39] | 2019 | America | ① | / | C57BL/6J mice | M or F | 16 | M:~34; F:~28 | IP. cecal plasma | Yes | / | No | Limb muscle | Ⅰ; Ⅱ | 4d, 2w, 1month | Food iintake | Muscle strength | CSA;Muscle fiber type | Body weight; Muscle weight | Mitochondrial structure and function; Inflammatory markers |
| Preau S[40] | 2019 | Britain | ① | Yes | Wistar rats | M | / | 300 | IP. zymosan | No | / | No | Limb muscle | Ⅱ | 2d, 7d, 14d | / | / | Muscle mass; Fiber  diameters; Atrophic genes or proteins; Autophagy | Body weight | / |
| Hill NE[41] | 2017 | Britain | ④ | Yes | Wistar rats | M | / | 264-333 | IP. zymosan | No | Con: n=5; Zymosan-vehicle: n=35; Zymosan-ghrelin: n=39 | No | Limb muscle | Ⅰ | 12d | Food iintake | Grip strength | Muscle mass; Muscle histology | Body weight | / |
| Witteveen E[42] | 2019 | Netherland | ③ | Yes | C57BL/6J mice | M | 8-10 | / | IN. S. pneumoniae | Yes | Con: n=8; Blank: n=4; Ceftriaxone was administered at 24 h: n=8; Ceftriaxone was administered at 48h: n = 8; Ceftriaxone was administered as soon as mice lost 10% of their body weight: n = 8 | No | Diaphragm； Limb muscle | Ⅰ | 120h | / | Grip strength;CMAP; Contractility | Myosin or/and actin protein | Body weight | / |
| Bloise FF[43] | 2016 | Netherland |  | Yes | C57Bl6 mice | F | 8-12 | / | IN. S. pneumoniae | No | / | No | Diaphragm； Limb muscle | / | 24h, 40h | / | / | Muscle fiber types; MHC | / | Mitochondrial function; Inflammatory markers |
| Disuse model | | | | | | | | | | | | | | | | | | | | |
| Kutz L[44] | 2023 | America | ③ | / | C57BL/6J mice | M or F | 8–10 | / | Tail suspension | No | / | No | Limb muscle | Ⅱ | 14d | / | / | Muscle mass | / | / |
| Yang J[45] | 2017 | China | ② | Yes | Sprague-Dawley rats | M | Adult | 240- 260 | Limb immobilization | No | Con: n=14; Sham: n=14; Immobilization: n=14; EA: n=14 | No | Limb muscle | Ⅰ; Ⅱ | 14d | / | CMAP; Muscle force | CSA; Muscle fiber size | Muscle weight | / |
| Onda A[46] | 2016 | Japan | ③ | / | C57BL/6 mice | M | 8–9 | / | Limb immobilization | No | Con: n=11; SW 3d: n=8; SWI 5d: n=12; SWI 10d: n=9 | No | Limb muscle | Ⅱ | 3d, 5, 10d | Food iintake | / | Atrophic genes or proteins | Body weight; Muscle weight | / |
| Aihara M[47] | 2017 | Japan | ③ | / | C57BL/6 mice | M | 10 | / | Limb immobilization | No | Con: n=5; Cast Immobilization: n=5; Velcro immobilization: n=5 | No | Limb muscle | Ⅱ | 2w | / | / | Muscle fiber diameter | Body weight; Muscle weight | / |
| ICU model | | | | | | | | | | | | | | | | | | | | |
| Mrozek S [48] | 2012 | French |  | / | C57/BL6 mice | M | 10-12 | 25-30 | MV | Yes | MV: n = 6; CPAP: n = 6; Con: n = 6 | No | Diaphragm; Limb muscle | Ⅰ | 6h | / | Muscle force | / | Body weigh | / |
| Guo HL [49] | 2011 | China |  | Yes | SD rats | M | / | / | MV | Yes | Con: n=7; CMV-18h: n=10; CMV-24h: n=14. | No | Diaphragm | Ⅰ; Ⅱ | 18h, 24h | / | / | CSA;Myofiber morphologyr; Muscle fiber type | Body weight | Mitochondrial structure |
| Matecki S [50] | 2017 | French |  | / | Piglets | / | / | 15-20 kg | MV | Yes | MV: n=5; Con: n=5 | No | Diaphragm | Ⅰ | 72h | / | Muscle force | / | / | / |
| Gayan-Ramirez G [51] | 2003 | Belgium |  | Yes | Wistar rats | M | / | / | MV | Yes | Con: n=10; SB: n=9 out of 26; Anesthetized and MV: n=12 out of 16 | No | Diaphragm； Limb muscle | Ⅰ; Ⅱ | 24h | / | Muscle force | CSA; Muscle fiber type ; | Body weight; Muscle weight | / |
| Radell P [52] | 2004 | America |  | / | Piglets | / | 2-4 | 23- 30 kg | MV | Yes | Con MV and sedation: n=7; Con animals ventilated for only 4–6h: n=5 | No | Diaphragm | / | 4-6h, 5d | / | / | CSA; Myosin or/and actin；Myofiber morphology | / | / |
| Radell PJ [53] | 2002 | America |  | / | Piglets | / | / | 20-25 kg | MV | Yes | Seven piglets were studied for 5 days. | No | Diaphragm | Ⅰ | 3-5d | / | CMAP | / | / | / |
| Tang H [54] | 2013 | America |  | / | FVB mice | M | / | 30 | MV | Yes | Con: n =9; MV: n=8 | No | Diaphragm； Limb muscle | Ⅱ | 18h | / | / | Myofiber morphology; Muscle fiber type; Atrophic genes or proteins; Autophagy; Apoptosis | Muscle weight | / |
| Shanely RA [55] | 2002 | America |  | Yes | SD rats | F | Adult | / | MV | Yes | 18h M: n = 16; Con (no MV): n = 16; Spontaneous breathing: n = 6 | No | Diaphragm; Limb muscle | Ⅱ | 18h | / | / | Muscle mass; Myofiber morphology; CSA | Body weight | / |
| Zambelli V [56] | 2019 | Italy | ④ | / | SD rats | / | 8 | 250-300 | MV | Yes | / | No | Diaphragm | Ⅰ; Ⅱ | 8h | / | Contractility | CSA; Atrophic genes or proteins | / | / |
| Llano-Diez M [57] | 2016 | Sweden |  | / | SD rats | F | / | 235-299 | A | Yes | SHAM: n=8; ICU: n=7 | No | Limb muscle | Ⅰ | 8 or 10d | / | Muscle force; Force generation | / | / | / |
| Addinsall AB [58] | 2022 | Sweden |  | / | SD rats | F | / | / | A | Yes | Con: n = 8; 8D ICU: n =7; 8DES ICU: n = 10 | No | Limb muscle | Ⅰ; Ⅱ | 8d | / | Muscle force | CSA; Myosin or/and actin protein; Atrophic genes or proteins | Body weight; Muscle weight | / |
| Corpeno R [59] | 2014 | Sweden |  | / | SD rats | F | / | / | A | Yes | Con: n=15; Anaesthetized and mechanically ventilated: n=46 | No | Diaphragm; Limb muscle | Ⅰ; Ⅱ | 0.25-4d, 5-8d, 9-14d | / | Muscle force | CSA; Myosin or/and actin protein; Atrophic genes or proteins | Body weight; Muscle weight | / |
| Llano-Diez M [60] | 2011 | Sweden | ③ | / | SD rats | F | / | / | A | Yes | Con: n=5; 23 experimental rats: 6h to 4 days: n = 13; From 5 to 8 days: n = 4; From 9 to 14 days n = 6 | No | Limb muscle | Ⅱ | 0.25-4d, 5-8d, 9-14d | / | / | Atrophic genes or proteins; Sarcomere protein | / | / |
| Akkad H [61] | 2014 | Sweden |  | / | SD rats | F | / | / | A | Yes | Four animals per: one con and three experimental: 0.25–4, 5–8 and 9–14day s | No | Masseter muscle | Ⅱ | 0.25-4d, 5-8d, 9-14d | / | / | Myofiber morphology; CSA; Myosin or/and actin protein; Atrophic genes or proteins; Autophagy | / | / |
| Renaud G [62] | 2013 | Sweden |  | / | SD rats | F | / | / | A | Yes | Con: n=15; Anaesthetized and mechanically ventilated: n=46 | No | Limb muscle | Ⅰ; Ⅱ | 0.25-4d, 5-8d, 9-14d | / | Muscle force | CSA; Myosin or/and actin protein; Atrophic genes or proteins | Body weight; Muscle weight | / |
| Ochala J [63] | 2011 | Sweden |  | / | SD rats | F | / | / | A | Yes | Sham-operated Con: n=15; Anaesthetized and mechanically ventilated: n=46. | No | Limb muscle | Ⅰ; Ⅱ | 0.25-4d, 5-8d, 9-14d | / | Force generating | Muscle fiber type; CSA; Myosin or/and actin protein; Atrophic genes or proteins | Body weight; Muscle weight | / |
| Banduseela VC [64] | 2009 | Sweden |  | / | Piglets | F | / | 26.5kg | B | Yes | Sixteen F domestic piglets were used in this study. | No | Limb muscle | / | 1d, 3d, 5d | / | Force generating | CSA; Atrophic genes or proteins; Sarcomere protein | / | / |
| Tang Z [65] | 2019 | China | ③ | Yes | SD rats | M | 10 | 393~421 | C | No | Six animals per: Con 3d, 6d,9d; Immobilization 3d, 6d, 9d; LPS 3d, 6d, 9d; LPS + immobilization 3d, 6d, 9d. | No | Diaphragm; Limb muscle | Ⅱ | 3d, 6d, 9d | / | / | Myofiber morphology; CSA; Atrophic genes or proteins | Body weight | / |
| Habr B [66] | 2020 | America |  | / | Wistar rats | M | 12 | / | C | No | n = 10 per: Sham; Sham HCT; SI; SI HCT. | No | Limb muscle | Ⅰ; Ⅱ | 5d | / | Muscle force; Grip strength | CSA; Muscle mass; Myosin or/and actin protein | Body weight | / |
| Jiang Y [67] | 2022 | China |  | / | C57BL/6J mice | M | 12 | 22-24 | C | Yes | Con: n=6; Sepsis: n=6; Sepsis + immobilization: n=6 | No | Limb muscle | Ⅰ; Ⅱ | 5d | / | Grip strength | Muscle fibe typer; Myofiber morphology; Potential biomarkers for ICU-AW; | Muscle weight | Mitochondrial structure and function; Inflammatory markers |
| Laitano O [68] | 2021 | Mexico | ③ | Yes | C57BL/6J mice | M | ~4 | / | C | Yes | SHAM+ normal ambulation: n = 6; SHAM+ hindlimb suspension: n = 6; CLP+ normal ambulation: n = 6; CLP+ hindlimb suspension: n = 8 | No | Limb muscle | Ⅰ; Ⅱ | 7d | / | Muscle force | Muscle mass; CSA | Body weight; Muscle weight | / |
| Grunow JJ [69] | 2023 | German |  | Yes | SD rats | M | / | / | C | Yes | Con: n=8; Sham-immobilization: n=25; Immobilization: n=26; Inflammation: n=35; Inflammation+ immobilization: n=37 | Yes | Limb muscle | Ⅱ | 0d, 4d, 8d, 12d | / | / | Muscle atrophy | Body weight; Muscle weight | / |
| Ackermann KA [70] | 2014 | America |  | Yes | Pigs | / | / | 40 ± 3 kg | D | Yes | Faecal peritonitis: n = 10; Non-septic Con: n = 10. | No | Limb muscle | Ⅰ | 6h, 17h, 27h | / | CMAP | / | / | / |
| Li LF[71] | 2021 | China |  | Yes | C57BL/6J mice | / | 6-8 | 20-25 | D | No | Eight animals per: non-ventilated Con wild-type mice with normal saline; Non-ventilated Con wild-type mice with LPS; VT 6 mL/kg wild-type mice with LPS; VT 10 mL/kg wild-type mice with normal saline; VT 10 mL/kg wild-type mice with LPS; VT 10 mL/kg HIF-1α−/− mice with LPS; VT 10 mL/kg wild-type mice after enoxaparin administration with LPS | No | Diaphragm | Ⅰ; Ⅱ | 8h | / | Muscle force | Myofiber morphology; Atrophic genes or proteins | / | Mitochondrial structure |
| Le Dinh M [72] | 2018 | French |  | Yes | Wistar rats | M | / | 450 - 600 | D | Yes | SV-LPS: n = 8; MV: n = 8; MV-LPS: n = 8; Con: n = 8 | No | Diaphragm | Ⅰ; Ⅱ | 12h | / | Muscle force; Force generating | CSA; Muscle fiber type | / | / |
| Yamada T [73] | 2018 | Japan |  | Yes | Wistar rats | M | 9 | / | Denervation + dexamethasone | No | Con: n = 12; Steroid-denervation: n = 12 | No | Limb muscle | Ⅰ; Ⅱ | 7d | / | Muscle force | Muscle fiber changes; Atrophic genes or proteins; Myosin or/and actin protein | Body weight; Muscle weight | / |
| Barnes BT [74] | 2015 | America |  | / | Wistar rats | M | Adult | 250-350 | Denervation + dexamethasone | Yes | Steroid-denervation: n=8; Con: n=9 | No | Limb muscle | Ⅱ | 7d, 7-10d | / | / | Muscle fiber changes; Apoptosis | Muscle weight | / |
| Shimatani T [75] | 2019 | Japan |  | Yes | Rabbit | / | / | / | MV+NMBA | Yes | Twenty Japanese white rabbits were randomly divided into four：no ventilation +NMBA; CMV; NAVA; PSV | No | Diaphragm | Ⅱ | 12h | / | / | Muscle fiber changes; CSA;  Apoptosis | / | / |
| Banduseela VC [76] | 2013 | Sweden |  | / | Pigs | F | / | 26.5 kg | E | Yes | ICU-1: n=4; CII: n=4 | No | Limb muscle | / | 5d | / | / | Sarcomere protein; Autophagy | / | / |
| Aare S[77] | 2011 | Sweden |  | / | Piglets | / | / | 26.9 kg | E | Yes | Five F domestic piglets were used in this study. | No | Masseter muscle; Limb muscle | Ⅰ; Ⅱ | 1d, 5d | / | Muscle force | CSA; Atrophic genes or proteins; Sarcomere protein; Muscle fiber type | / | / |
| Ochala J [78] | 2011 | Sweden |  | Yes | Piglets | F | / | 23-30kg | E | Yes | MV: n=4; Sepsis: n=4; NMBA: n=3; CS: n=3; ALL: n=4 | No | Limb muscle | Ⅰ; Ⅱ | 5d | / | Muscle force; CMAP | CSA; Myosin or/and actin protein | / | / |
| Ochala J [79] | 2011 | Sweden |  | Yes | Piglets | F | / | 23-30kg | E | Yes | CTL n=4; MV; n=4; Sepsis n=4; NMBA n=3; CS n=3; ALL: n=4 | No | Diaphragm | Ⅰ; Ⅱ | 5d | / | Muscle force | CSA; Myosin or/and actin protein; Muscle fiber type | / | / |
| Other models | | | | | | | | | | | | | | | | | | | | |
| Liao WC [80] | 2018 | China |  | Yes | SD rats | M | / | 240–340 | Pancreatitis + abdominal hypertension | No | Con: n = 5; SAP: n = 5; SAP+IAH: n = 5; SAP+IAH+SS-31: n = 5. | No | Diaphragm | Ⅰ; Ⅱ | 12h | / | Contractility | Myofiber morphology | / | / |
| Radigan KA[81] | 2019 | America |  | / | C57BL/6 mice | M | 12-16 | / | Viral infection | No | / | No | Limb muscle | Ⅰ; Ⅱ | 7d | / | Grip strength | CSA; Atrophic genes or proteins | Body weight; Muscle weight | / |
| Wu X[82] | 2010 | America |  | Yes | SD rats | M | / | ~300 | Burned | Yes | Sham burned: n=32; burned: 3d: n=8; 7d: n=8; 14d: n=8; 21d: n=8 | No | Limb muscle | Ⅰ | 3d. 7d, 14d, 21d | / | Muscle force | / | Body weight; Muscle weight | / |
| [Yuko Ono](https://pubmed.ncbi.nlm.nih.gov/?term=Ono%20Y%5bAuthor%5d) [83] | 2020 | Japan | ④ | / | C57BL/6 mice | M | 12-16 | 24-29 g | Burned | Yes | / | No | Limb muscle | Ⅰ; Ⅱ | 3d | Food iintake | Grip strength | Myofiber morphology; Atrophic genes or proteins; Protein hydrolysis pathway | Body weight; Muscle weight | / |
| Callahan LA[84] | 2014 | America |  | / | SD rats | M | Adult | 250 and 350 | Induction of hyperglycaemia using streptozotocin | Yes | / | No | Diaphragm | Ⅰ | 2w | / | Muscle force | / | Body weight; Muscle weight | / |

Note: ①: Exploring the pathogenesis; ②: Exploring physical intervention mechanisms; ③: Constructing animal model; ④: Exploring mechanisms of pharmacological or others intervention.

A: sedation or anaesthesia + mechanical ventilation+ Neuromuscular blocking agent (NMBA); ARDS: Acute Respiratory Distress Syndrome; B: mechanical ventilation+ immobilization; C: sepsis+ immobilization; CLP: cecal ligation perforation; CMV: controlled mechanical ventilation; CMAP: compound muscle action potential; Con: control; CPAP: continuous positive airway pressure; D: sepsis+ mechanical ventilation; E: Sedation or anaesthesia + mechanical ventilation+ neuromuscular blocking agent (NMBA) + sepsis + corticosteroids; IAH: intra-abdominal hypertension; IP: intraperitoneal injection; IV: intravenous injection; IN: intranasal inoculation; LPS: Lipopolysaccharide; LY: TGF-β pathway inhibitors; SD rat: Sprague-Dawley rats; M: male; MHC: myosin skeletal heavy chain; MV: mechanical ventilation; NAVA: neurally adjusted ventilatory assist; F: female; PSV: pressure support ventilation; SAP: severe acute pancreatitis; SG: sepsis+ glutamine; SB: spontaneous breathing; SE: sepsis +leucine; SLG: sepsis + glutamine + leucine; WT: wildlife

Ⅰ: Indicators of muscle weakness; Ⅱ: Indicators of muscle atrophy; Ⅲ: Decreased excitability

/: Not describe;

**Reference**

1. Nardelli P, Vincent JA, Powers R, Cope TC, Rich MM. (2016). Reduced motor neuron excitability is an important contributor to weakness in a rat model of sepsis. Exp Neurol. 282:1-8; doi: 10.1016/j.expneurol.2016.04.020.

2. Zhang JY, Wu J, Li ST.Gong Y. (2016). [Lowered sarcoendoplasmic reticulum calcium uptake and diaphragmatic SERCA1 expression contribute to diaphragmatic contractile and relaxation dysfunction in septic rats]. Nan Fang Yi Ke Da Xue Xue Bao. 37(4):438-443; doi: 10.3969/j.issn.1673-4254.2017.04.03.

3. Goossens C, Weckx R, Derde S, Van Helleputte L, Schneidereit D, Haug M, et al. (2021). Impact of prolonged sepsis on neural and muscular components of muscle contractions in a mouse model. J Cachexia Sarcopenia Muscle. 12(2):443-455; doi: 10.1002/jcsm.12668.

4. Jude B, Tissier F, Dubourg A, Droguet M, Castel T, Léon K, et al. (2020). TGF-β Pathway Inhibition Protects the Diaphragm From Sepsis-Induced Wasting and Weakness in Rat. Shock. 53(6):772-778; doi: 10.1097/shk.0000000000001

5. Alamdari N, Toraldo G, Aversa Z, Smith I, Castillero E, Renaud G, et al . (2012). Loss of muscle strength during sepsis is in part regulated by glucocorticoids and is associated with reduced muscle fiber stiffness. Am J Physiol Regul Integr Comp Physiol. 303(10):R1090-1099; doi: 10.1152/ajpregu.00636.2011.

6. Liu H, Pan D, Li P, Wang D, Xia B, Zhang R, et al. (2023). Loss of ZBED6 Protects Against Sepsis-Induced Muscle Atrophy by Upregulating DOCK3-Mediated RAC1/PI3K/AKT Signaling Pathway in Pigs. Adv Sci (Weinh). 10(29):e2302298; doi: 10.1002/advs.202302298.

7. Zheng Y, Dai H, Chen R, Zhong Y, Zhou C, Wang Y, et al. (2023). Endoplasmic reticulum stress promotes sepsis-induced muscle atrophy via activation of STAT3 and Smad3. J Cell Physiol. 238(3):582-596; doi: 10.1002/jcp.30950.

8. Weckx R, Goossens C, Derde S, Pauwels L, Vander Perre S, Van den Berghe G, et al. (2022). Efficacy and safety of ketone ester infusion to prevent muscle weakness in a mouse model of sepsis-induced critical illness. Sci Rep. 12(1):10591; doi:10.1038/s41598-022-14961-w.

9. Schmitt RE, Dasgupta A, Arneson-Wissink PC, Datta S, Ducharme AM. Doles JD. (2023). Muscle stem cells contribute to long-term tissue repletion following surgical sepsis. J Cachexia Sarcopenia Muscle. 14(3):1424-1440; doi: 10.1002/jcsm.13214.

10. Crowell KT.Lang CH. (2021). Contractility and Myofibrillar Content in Skeletal Muscle are Decreased During Post-Sepsis Recovery, But Not During the Acute Phase of Sepsis. Shock. 55(5):649-659; doi: 10.1097/shk.0000000000001555.

11. Rocheteau P, Chatre L, Briand D, Mebarki M, Jouvion G, Bardon J, et al. (2015). Sepsis induces long-term metabolic and mitochondrial muscle stem cell dysfunction amenable by mesenchymal stem cell therapy. Nat Commun. 6:10145; doi: 10.1038/ncomms10145.

12. Vankrunkelsven W, Derde S, Gunst J, Vander Perre S, Declerck E, Pauwels L, et al. (2022). Obesity attenuates inflammation, protein catabolism, dyslipidaemia, and muscle weakness during sepsis, independent of leptin. J Cachexia Sarcopenia Muscle. 13(1):418-433; doi: 10.1002/jcsm.12904.

13. Cao YY, Wang Z, Yu T, Zhang Y, Wang ZH, Lu ZM, et al. (2021). Sepsis induces muscle atrophy by inhibiting proliferation and promoting apoptosis via PLK1-AKT signalling. J Cell Mol Med. 25(20):9724-9739; doi: 10.1111/jcmm.16921.

14. Wang J. Wu T. (2020). Testosterone improves muscle function of the extensor digitorum longus in rats with sepsis. Bosci Rep. 40(2):BSR20193342; doi: 10.1042/bsr20193342.

15. Hou YC, Wu JM, Chen KY, Wu MH, Yang PJ, Lee PC, et al. (2023). Glutamine and leucine administration attenuates muscle atrophy in sepsis. Life Sci. 314:121327; doi: 10.1016/j.lfs.2022.121327.

16. Hou YC, Pai MH, Wu JM, Yang PJ, Lee PC, Chen KY, et al. (2021). Protective Effects of Glutamine and Leucine Supplementation on Sepsis-Induced Skeletal Muscle Injuries. Int J Mol Sci. 22(23):13003; doi: 10.3390/ijms222313003.

17. Li X, Sun B, Li J, Ye W, Li M, Guan F, et al. (2023). SEPSIS LEADS TO IMPAIRED MITOCHONDRIAL CALCIUM UPTAKE AND SKELETAL MUSCLE WEAKNESS BY REDUCING THE MICU1:MCU PROTEIN RATIO. S hock. 60(5):698-706; doi: 10.1097/shk.0000000000002221.

18. Chen J, Chen XY, Cong XX, Wang S, Xu SB, Sun YT, et al. (2023). CELLULAR SENESCENCE IMPLICATED IN SEPSIS-INDUCED MUSCLE WEAKNESS AND AMELIORATED WITH METFORMIN. Shock. 59(4):646-656; doi: 10.1097/shk.0000000000002086.

19. Hahn A, Kny M, Pablo-Tortola C, Todiras M, Willenbrock M, Schmidt S, et al. (2020). Serum amyloid A1 mediates myotube atrophy via Toll-like receptors. J Cachexia Sarcopenia Muscle. 11(1):103-119; doi: 10.1002/jcsm.12491.

20. Nardelli P, Khan J, Powers R, Cope TC. Rich MM. (2013). Reduced motoneuron excitability in a rat model of sepsis. J Neurophysiol. 109(7):1775-1781; doi: 10.1152/jn.00936.2012.

21. Zanders L, Kny M, Hahn A, Schmidt S, Wundersitz S, Todiras M, et al. (2022). Sepsis induces interleukin 6, gp130/JAK2/STAT3, and muscle wasting. J Cachexia Sarcopenia Muscle. 13(1):713-727; doi: 10.1002/jcsm.12867.

22. Wang C, Liu Y, Zhang Y, Wang D, Xu L, Li Z, et al. (2023). Targeting NAT10 protects against sepsis-induced skeletal muscle atrophy by inhibiting ROS/NLRP3. Life Sci. 330:121948; doi: 10.1016/j.lfs.2023.121948.

23. Supinski GS, Wang L, Schroder EA, Callahan LAP. (2020). Taurine administration ablates sepsis induced diaphragm weakness. Respir Physiol Neurobiol. 271:103289; doi: 10.1016/j.resp.2019.103289.

24. Supinski GS, Wang L, Song XH, Moylan JS, Callahan LA. (2014). Muscle-specific calpastatin overexpression prevents diaphragm weakness in cecal ligation puncture-induced sepsis. J Appl Physiol (1985). 117(8):921-929; doi: 10.1152/japplphysiol.00975.2013.

25. Cankayali I, Dogan YH, Solak I, Demirag K, Eris O, Demirgoren S, et al. (2007). Neuromuscular deterioration in the early stage of sepsis in rats. Crit Care. 11(1):R1; doi: 10.1186/cc5139.

26. Rossignol B, Gueret G, Pennec JP, Morel J, Rannou F, Giroux-Metges MA, et al. (2008). Effects of chronic sepsis on contractile properties of fast twitch muscle in an experimental model of critical illness neuromyopathy in the rat. Crit Care Med. 36(6):1855-1863; doi: 10.1097/CCM.0b013e318176106b.

27. Supinski GS, Wang L, Schroder EA, Callahan LAP. (2020). SS31, a mitochondrially targeted antioxidant, prevents sepsis-induced reductions in diaphragm strength and endurance. J Appl Physiol (1985). 128(3):463-472; doi: 10.1152/japplphysiol.00240.2019.

28. Liu L, Min S, Li W, Wei K, Luo J, Wu G, et al. (2014). Pharmacodynamic changes with vecuronium in sepsis are associated with expression of α 7- and γ -nicotinic acetylcholine receptor in an experimental rat model of neuromyopathy. Br J Anaesth. 112(1):159-168; doi: 10.1093/bja/aet253.

29. Vankrunkelsven W, Thiessen S, Derde S, Vervoort E, Derese I, Pintelon I, et al. (2023). Development of muscle weakness in a mouse model of critical illness: does fibroblast growth factor 21 play a role? Skelet Muscle. 13(1):12; doi: 10.1186/s13395-023-00320-4.

30. Mengmeng W. (2017). Alterations in calcium concentration and DHPR, RyR on diaphragm dysfunction in a rat model of sepsis. Doctor. China Medical University.

31. Jiao GY, Hao LY, Gao CE, Chen L, Sun XF, Yang HL, et al. (2013). Reduced DHPRα1S and RyR1 expression levels are associated with diaphragm contractile dysfunction during sepsis. Muscle Nerve. 48(5):745-751; doi: 10.1002/mus.23805.

32. Al-Nassan S, Fujino H. (2018). Exercise preconditioning attenuates atrophic mediators and preserves muscle mass in acute sepsis. Gen Physiol Biophys. 37(4):433-441; doi: 10.4149/gpb_2018001.

33. Liu L, Li TM, Liu XR, Bai YP, Li J, Tang N, et al. (2019). MicroRNA-140 inhibits skeletal muscle glycolysis and atrophy in endotoxin-induced sepsis in mice via the WNT signaling pathway. Am J Physiol Cell Physiol. 317(2):C189-c199; doi: 10.1152/ajpcell.00419.2018.

34. Ono Y, Maejima Y, Saito M, Sakamoto K, Horita S, Shimomura K,et al. TAK-242, a specific inhibitor of Toll-like receptor 4 signalling, prevents endotoxemia-induced skeletal muscle wasting in mice. Sci Rep. 2020 Jan 20;10(1):694.

35. Frick CG, Fink H, Gordan ML, Eckel B, Martyn JA, Blobner M. (2008). Chronic Escherichia coli infection induces muscle wasting without changing acetylcholine receptor numbers. Intensive Care Med. 34(3):561-567; doi: 10.1007/s00134-007-0852-3.

36. Witteveen E, Hoogland IC, Wieske L, Weber NC, Verhamme C, Schultz MJ, et al. (2016). Assessment of intensive care unit-acquired weakness in young and old mice: An E. coli septic peritonitis model. Muscle Nerve. 53(1):127-133; doi: 10.1002/mus.24711..

37. Pierre A, Bourel C, Favory R, Brassart B, Wallet F, Daussin FN, et al. (2023). Sepsis-like Energy Deficit Is Not Sufficient to Induce Early Muscle Fiber Atrophy and Mitochondrial Dysfunction in a Murine Sepsis Model. Biology (Basel). 12(4):529; doi: 10.3390/biology12040529.

38. Nakanishi N, Ono Y, Miyazaki Y, Moriyama N, Fujioka K, Yamashita K, et al. (2022). Sepsis causes neutrophil infiltration in muscle leading to muscle atrophy and weakness in mice. Front Immunol. 13:950646; doi: 10.3389/fimmu.2022.950646.

39. Owen AM, Patel SP, Smith JD, Balasuriya BK, Mori SF, Hawk GS, et al. (2019). Chronic muscle weakness and mitochondrial dysfunction in the absence of sustained atrophy in a preclinical sepsis model. Elife. 8:e49920; doi: 10.7554/eLife.49920.

40. Preau S, Ambler M, Sigurta A, Kleyman A, Dyson A, Hill NE, et al. (2019). Protein recycling and limb muscle recovery after critical illness in slow- and fast-twitch limb muscle. Am J Physiol Regul Integr Comp Physiol. 316(5):R584-r593; doi: 10.1152/ajpregu.00221.2018.

41. Hill NE, Murphy KG, Saeed S, Phadke R, Chambers D, Wilson DR, et al. (2017). Impact of ghrelin on body composition and muscle function in a long-term rodent model of critical illness. PLoS One. 12(8):e0182659; doi: 10.1371/journal.pone.0182659.

42. Witteveen E, Wieske L, Manders E, Verhamme C, Ottenheijm CAC, Schultz MJ, et al. (2019). Muscle weakness in a S. pneumoniae sepsis mouse model. Ann Transl Med. 7(1):9; doi: 10.21037/atm.2018.12.45.

43. Bloise FF, van der Spek AH, Surovtseva OV, Ortiga-Carvalho TM, Fliers E.Boelen A. (2016). Differential Effects of Sepsis and Chronic Inflammation on Diaphragm Muscle Fiber Type, Thyroid Hormone Metabolism, and Mitochondrial Function. Thyroid. 26(4):600-609; doi: 10.1089/thy.2015.0536.

44. Kutz L, Zhou T, Chen Q, Zhu H. (2023). A Surgical Approach to Hindlimb Suspension: A Mouse Model of Disuse-Induced Atrophy. Methods Mol Biol. 2597:1-9; doi: 10.1007/978-1-0716-2835-5_1.

45. Yang J, Min S, Xie F, Chen J, Hao X, Ren L. (2017). Electroacupuncture alleviates neuromuscular dysfunction in an experimental rat model of immobilization. Oncotarget. 8(49):85537-85548; doi: 10.18632/oncotarget.20246.

46. Onda A, Kono H, Jiao Q, Akimoto T, Miyamoto T, Sawada Y, et al. (2016). New mouse model of skeletal muscle atrophy using spiral wire immobilization. Muscle Nerve. 54(4):788-791; doi: 10.1002/mus.25202.

47. Aihara M, Hirose N, Katsuta W, Saito F, Maruyama H, Hagiwara H. (2017). A new model of skeletal muscle atrophy induced by immobilization using a hook-and-loop fastener in mice. J Phys Ther Sci. 29(10):1779-1783; doi: 10.1589/jpts.29.1779.

48. Mrozek S, Jung B, Petrof BJ, Pauly M, Roberge S, Lacampagne A, et al. (2012). Rapid onset of specific diaphragm weakness in a healthy murine model of ventilator-induced diaphragmatic dysfunction. Anesthesiology. 117(3):560-567; doi: 10.1097/ALN.0b013e318261e7f8.

49. Hongli G, Xiaohong W, Shaolin M, Xiaoping C, Xiaoping Z. (2012). Effects of mechanical

ventilation on morphological properties and myosin heavy chain of diaphragm fibers in rats. %J Journal of Xi'an Jiaotong University (Medical Edition). 33, 324-328.

50. Matecki S, Jung B, Saint N, Scheuermann V, Jaber S, Lacampagne A. (2017). Respiratory muscle contractile inactivity induced by mechanical ventilation in piglets leads to leaky ryanodine receptors and diaphragm weakness. J Muscle Res Cell Motil. 38(1):17-24; doi: 10.1007/s10974-017-9464-x.

51. Radell PJ, Remahl S, Nichols DG, Eriksson LI. (2002). Effects of prolonged mechanical ventilation and inactivity on piglet diaphragm function. Intensive Care Med. 28(3):358-364; doi: 10.1007/s00134-002-1207-8.

52. Radell P, Edström L, Stibler H, Eriksson LI.Ansved T. (2004). Changes in diaphragm structure following prolonged mechanical ventilation in piglets. Acta Anaesthesiol Scand. 48(4):430-437; doi: 10.1111/j.1399-6576.2004.00352.x.

53. Radell PJ, Remahl S, Nichols DG, Eriksson LI. (2002). Effects of prolonged mechanical ventilation and inactivity on piglet diaphragm function. Intensive Care Med. 28(3):358-364; doi: 10.1007/s00134-002-1207-8.

54. Tang H, Lee M, Khuong A, Wright E, Shrager JB. (2013). Diaphragm muscle atrophy in the mouse after long-term mechanical ventilation. Muscle Nerve. 48(2):272-278; doi: 10.1002/mus.23748.

55. Shanely RA, Zergeroglu MA, Lennon SL, Sugiura T, Yimlamai T, Enns D, et al. (2002). Mechanical ventilation-induced diaphragmatic atrophy is associated with oxidative injury and increased proteolytic activity. Am J Respir Crit Care Med. 166(10):1369-1374; doi: 10.1164/rccm.200202-088OC.

56. Zambelli V, Sigurtà A, Rizzi L, Zucca L, Delvecchio P, Bresciani E, et al. (2019). Angiotensin-(1-7) exerts a protective action in a rat model of ventilator-induced diaphragmatic dysfunction. Intensive Care Med Exp. 7(1):8; doi: 10.1186/s40635-018-0218-x.

57. Llano-Diez M, Cheng AJ, Jonsson W, Ivarsson N, Westerblad H, Sun V, et al. (2016). Impaired Ca(2+) release contributes to muscle weakness in a rat model of critical illness myopathy. Crit Care. 20(1):254; doi: 10.1186/s13054-016-1417-z.

58. Addinsall AB, Cacciani N, Backéus A, Hedström Y, Shevchenko G, Bergquist J, et al. (2022). Electrical stimulated GLUT4 signalling attenuates critical illness-associated muscle wasting. J Cachexia Sarcopenia Muscle. 13(4):2162-2174; doi: 10.1002/jcsm.12978.

59. Corpeno R, Dworkin B, Cacciani N, Salah H, Bergman HM, Ravara B, et al. (2014). Time course analysis of mechanical ventilation-induced diaphragm contractile muscle dysfunction in the rat. J Physiol. 592(17):3859-3880; doi: 10.1113/jphysiol.2014.277962.

60. Llano-Diez M, Gustafson AM, Olsson C, Goransson H, Larsson L. (2011). Muscle wasting and the temporal gene expression pattern in a novel rat intensive care unit model. BMC Genomics. 12:602; doi: 10.1186/1471-2164-12-602.

61. Akkad H, Corpeno R, Larsson L. (2014). Masseter muscle myofibrillar protein synthesis and degradation in an experimental critical illness myopathy model. PLoS One. 9(4):e92622; doi: 10.1371/journal.pone.0092622.

62. Renaud G, Llano-Diez M, Ravara B, Gorza L, Feng HZ, Jin JP, et al. (2013). Sparing of muscle mass and function by passive loading in an experimental intensive care unit model. J Physiol. 591(5):1385-1402; doi: 10.1113/jphysiol.2012.248724.

63. Ochala J, Gustafson AM, Diez ML, Renaud G, Li M, Aare S, et al. (2011). Preferential skeletal muscle myosin loss in response to mechanical silencing in a novel rat intensive care unit model: underlying mechanisms. J Physiol. 589(Pt 8):2007-2026; doi: 10.1113/jphysiol.2010.202044.

64. Banduseela VC, Ochala J, Chen YW, Göransson H, Norman H, Radell P, et al. (2009). Gene expression and muscle fiber function in a porcine ICU model. Physiol Genomics. 39(3):141-159; doi: 10.1152/physiolgenomics.00026.2009.

65. Zhang T, Zhaoxia X, Ya L, Zhongli Z, Yuxi S, Chaox S, et al. (2019). Study on the establishmet of ICU-acquired weakness model in rats with sepsis. %J South West Defence Medicine. 29, 633-636.

66. Habr B, Saliba Y, Hajal J, Smayra V, Riachy M, Fares N. (2020). Hydrocortisone mitigates ICU-AW by fine-tuning of muscle atrophic and hypertrophic signaling pathways in a sepsis model with limb immobilization. L ife Sci. 261:118366; doi: 10.1016/j.lfs.2020.118366.

67. Jiang Y, Wei Q, Liu W, Chen Q, Chen X, Yuan Z, et al. (2022). Exploring the Muscle Metabolomics in the Mouse Model of Sepsis-Induced Acquired Weakness. Evid Based Complement Alternat Med. 2022:6908488; doi: 10.1155/2022/6908488.

68. Laitano O, Pindado J, Valera I, Spradlin RA, Murray KO, Villani KR, et al. (2021). The impact of hindlimb disuse on sepsis-induced myopathy in mice. Physiol Rep. 9(14):e14979; doi: 10.14814/phy2.14979.

69. Grunow JJ, Gan T, Lewald H, Martyn JAJ, Blobner M, Schaller SJ. (2023). Insulin signaling in skeletal muscle during inflammation and/or immobilisation. Intensive Care Med Exp. 11(1):16; doi: 10.1186/s40635-023-00503-9.

70. Ackermann KA, Bostock H, Brander L, Schröder R, Djafarzadeh S, Tuchscherer D, et al.(2014). Early changes of muscle membrane properties in porcine faecal peritonitis. Crit Care. 18(4):484; doi: 10.1186/s13054-014-0484-2.

71. Li LF, Yu CC, Huang HY, Wu HP, Chu CM, Huang CY, et al. (2021). Suppression of Hypoxia-Inducible Factor 1 α by Low-Molecular-Weight Heparin Mitigates Ventilation-Induced Diaphragm Dysfunction in a Murine Endotoxemia Model. Int J Mol Sci. 22(4):1702; doi: 10.3390/ijms22041702.

72. Le Dinh M, Carreira S, Obert J, Gayan-Ramirez G, Riou B, Beuvin M, et al. (2018). Prolonged mechanical ventilation worsens sepsis-induced diaphragmatic dysfunction in the rat. PLoS One. 13(8):e0200429; doi: 10.1371/journal.pone.0200429.

73. Yamada T, Himori K, Tatebayashi D, Yamada R, Ashida Y, Imai T, et al. (2018). Electrical Stimulation Prevents Preferential Skeletal Muscle Myosin Loss in Steroid-Denervation Rats. Front Physiol. 9:1111; doi: 10.3389/fphys.2018.01111.

74. Barnes BT, Confides AL, Rich MM, Dupont-Versteegden EE. (2015). Distinct muscle apoptotic pathways are activated in muscles with different fiber types in a rat model of critical illness myopathy. J Muscle Res Cell Motil. 36(3):243-253; doi: 10.1007/s10974-015-9410-8.

75. Barnes BT, Confides AL, Rich MM, Dupont-Versteegden EE. (2015). Distinct muscle apoptotic pathways are activated in muscles with different fiber types in a rat model of critical illness myopathy. J Muscle Res Cell Motil. 36(3):243-253; doi: 10.1007/s10974-015-9410-8.

76. Banduseela VC, Chen YW, Kultima HG, Norman HS, Aare S, Radell P, et al. (2013). Impaired autophagy, chaperone expression, and protein synthesis in response to critical illness interventions in porcine skeletal muscle. Physiol Genomics. 45(12):477-486; doi: 10.1152/physiolgenomics.00141.2012.

77. Aare S, Ochala J, Norman HS, Radell P, Eriksson LI, Göransson H, et al. (2011). Mechanisms underlying the sparing of masticatory versus limb muscle function in an experimental critical illness model. Physiol Genomics. 43(24):1334-1350; doi: 10.1152/physiolgenomics.00116.2011.

78. Ochala J, Ahlbeck K, Radell PJ, Eriksson LI.Larsson L. (2011). Factors underlying the early limb muscle weakness in acute quadriplegic myopathy using an experimental ICU porcine model. PLoS One. 6(6):e20876; doi: 10.1371/journal.pone.0020876.

79. Ochala J, Renaud G, Llano Diez M, Banduseela VC, Aare S, Ahlbeck K, et al. (2011). Diaphragm muscle weakness in an experimental porcine intensive care unit model. PLoS One. 6(6):e20558; doi: 10.1371/journal.pone.0020558.

80. Liao WC, Chen YH, Li HY, Wang TT, Lan P, Pan KH, et al. (2018). Diaphragmatic dysfunction in sepsis due to severe acute pancreatitis complicated by intra-abdominal hypertension. J Int Med Res. 46(4):1349-1357; doi: 10.1177/0300060517747163.

81. Radigan KA, Nicholson TT, Welch LC, Chi M, Amarelle L, Angulo M, et al. (2019). Influenza A Virus Infection Induces Muscle Wasting via IL-6 Regulation of the E3 Ubiquitin Ligase Atrogin-1. J Immunol. 202(2):484-493; doi: 10.4049/jimmunol.1701433.

82. Wu X, Wolf SE, Walters TJ. (2010). Muscle contractile properties in severely burned rats. Burns. 36(6):905-911; doi: 10.1016/j.burns.2010.02.003.

83. Ono Y, Saito M, Sakamoto K, Maejima Y, Misaka S, Shimomura K, Nakanishi N, Inoue S, Kotani J. C188-9, a specific inhibitor of STAT3 signaling, prevents thermal burn-induced skeletal muscle wasting in mice. Front Pharmacol. 2022 Dec 16;13:1031906.

84. Callahan LA, Supinski GS. (2014). Hyperglycemia-induced diaphragm weakness is mediated by oxidative stress. Crit Care. 18(3):R88; doi: 10.1186/cc13855.
